# Mettl14-mediated m6A modification is essential for visual function and retinal photoreceptor survival

**DOI:** 10.1186/s12915-022-01335-x

**Published:** 2022-06-13

**Authors:** Yeming Yang, Ping Shuai, Xiao Li, Kuanxiang Sun, Xiaoyan Jiang, Wenjing Liu, Weidong Le, Haisong Jiang, Yuping Liu, Xianjun Zhu

**Affiliations:** 1 Health Management Center, Sichuan Provincial People’s Hospital, University of Electronic Science and Technology of China, Chengdu, 61007 Sichuan China; 2 The Sichuan Provincial Key Laboratory for Human Disease Gene Study, Center for Medical Genetics, Sichuan Provincial People’s Hospital, University of Electronic Science and Technology of China, Chengdu, 610072 Sichuan China; 3 Key Laboratory of Tibetan Medicine Research, Chinese Academy of Sciences and Qinghai Provincial Key Laboratory of Tibetan Medicine Research, Northwest Institute of Plateau Biology, Xining, 810008 Qinghai China; 4 Institute of Neurology, Sichuan Provincial People’s Hospital, University of Electronic Science and Technology of China, Chengdu, 610072 Sichuan China; 5 Department of Neurology, Sichuan Provincial People’s Hospital, Chengdu, Sichuan China; 6 Research Unit for Blindness Prevention of the Chinese Academy of Medical Sciences (2019RU026), Sichuan Academy of Medical Sciences and Sichuan Provincial People’s Hospital, Chengdu, 610072 Sichuan China; 7 Henan Eye Institute, Henan Eye Hospital, People’s Hospital of Zhengzhou University, Henan Provincial People’s Hospital, Zhengzhou, 450003 Henan China

**Keywords:** N6-methyladenosine, METTL14, Photoreceptor degeneration, Phototransduction, Ciliogenesis

## Abstract

**Background:**

As the most abundant epigenetic modification of eukaryotic mRNA, N6-methyladenosine (m6A) modification has been shown to play a role in mammalian nervous system development and function by regulating mRNA synthesis and degeneration. However, the role of m6A modification in retinal photoreceptors remains unknown.

**Results:**

We generated the first retina-specific *Mettl14*-knockout mouse models using the Rho-Cre and HRGP-Cre lines and investigated the functions of *Mettl14* in retinal rod and cone photoreceptors. Our data showed that loss of *Mettl14* in rod cells causes a weakened scotopic photoresponse and rod degeneration. Further study revealed the ectopic accumulation of multiple outer segment (OS) proteins in the inner segment (IS). Deficiency of *Mettl14* in cone cells led to the mislocalization of cone opsin proteins and the progressive death of cone cells. Moreover, *Mettl14* depletion resulted in drastic decreases in METTL3/WTAP levels and reduced m6A methylation levels. Mechanistically, transcriptomic analyses in combination with MeRIP-seq illustrated that m6A depletion via inactivation of *Mettl14* resulted in reduced expression levels of multiple phototransduction- and cilium-associated genes, which subsequently led to compromised ciliogenesis and impaired synthesis and transport of OS-residing proteins in rod cells.

**Conclusions:**

Our data demonstrate that *Mettl14* plays an important role in regulating phototransduction and ciliogenesis events and is essential for photoreceptor function and survival, highlighting the importance of m6A modification in visual function.

**Supplementary Information:**

The online version contains supplementary material available at 10.1186/s12915-022-01335-x.

## Background

Retinal photoreceptors mediate vision formation by converting light stimuli from the environment into neural impulses to be sent to the brain. Photoreceptors are classified into two types according to differences in morphology and function: rods for dim light vision and cones for daylight and high-acuity vision. Both rods and cones are further subdivided in an inner segment (IS) and an outer segment (OS). The OS, where the phototransduction machinery resides, contains high concentrations of phototransduction proteins such as the G-protein transducin and visual pigments within membrane-bound discs, which are responsible for detecting light signals and transducing photons into electrical signals through the phototransduction cascade [1]. The IS houses the biosynthetic machinery and energy sources needed to produce and assemble newly synthesized phototransduction proteins and their associated membranes. The OS and IS of photoreceptors are connected by a highly modified primary cilium, also known as the connecting cilium (CC), which is homologous to the transition zone (TZ) of the primary cilium [2]. The CC is a microtubule-based structure consisting of an axoneme with nine doublet microtubules that are nucleated by nine triplet microtubules at the apical surface of the IS, termed the basal body (BB). The axoneme extends into the photoreceptor OS, where it is converted into singlet microtubules, and occupies 60 ~ 100% of the OS length in rods and 100% of the OS length in cones [3].

In photoreceptors, the CC serves as a conduit to mediate the transport of photopigment molecules, phototransduction proteins, and phospholipid components of the discs from the IS to the OS [2, 4]. Ciliary dysfunction causes disturbances in the trafficking and docking of specific proteins involved in the development and maintenance of the photoreceptor OS, which ultimately leads to photoreceptor degeneration. A number of ciliary defects are caused by dysfunction of the initial ciliogenesis process. In photoreceptors, ciliogenesis proceeds in a stepwise manner: the BB is derived from the mother centriole and docks on the apical membrane through the function of distal appendages (DAPs), initiating outgrowth of the axoneme [5]. This process depends on multiple molecules residing in the centriole/BB. For instance, CEP164, RAB8/CEP290, ODF2, and pericentrin are required for ciliary assembly, while IFT, CEP57, CEP290, CEP97, CEP131, CEP152, and ALMS1 mainly promote cilium elongation [6]; however, how this process is regulated remains largely unknown. Defects in ciliogenesis and cilium length control in humans have been implicated in a variety of inherited retinal dystrophies (IRDs), which usually present as isolated diseases or as a feature of complex multiorgan syndromic disorders termed ciliopathies, such as Joubert syndrome, Leber congenital amaurosis, Bardet-Biedl syndrome, and Usher syndrome type 2A [7]. These IRDs are genetically heterogeneous and involve mutations in a large number of genes [8]. An increasing number of causative genes of IRDs have been identified, but they can only explain approximately 60% of cases. The precise molecular mechanisms are still largely unknown.

In addition to classical Mendelian inheritance, epigenetic regulation has been found to play a role in the regulation of numerous cellular activities and pathological processes [9]. N6-methyladenosine (m6A), the most abundant modification of eukaryotic mRNA[10], participates in multiple biological processes by regulating mRNA metabolism, including alternative splicing, stability, nuclear export, degradation, and translation [11]. In mammalian cells, approximately 0.1 to 0.4% of adenines (A) exhibit m6A methylation, with an average of 3 to 5 methylated sites in each mRNA [12, 13]. m6A modifications generally occur in the RRACH (R = G or A, H = A or C or U) motif and mainly reside near stop codons and the 3′UTR of mRNAs [10, 14]. In addition, m6A modification has been shown to be a dynamic and reversible modification that is deposited by a multicomponent complex with methyltransferase-like (METTL)-3/14 and WTAP as the key methyltransferases and erased by the demethylases FTO and ALKBH5 [15–17]. During the methylation process, METTL3 acts as the catalytic component of the complex, while METTL14 functions in structural stabilization and RNA substrate recognition [18, 19]. After transcripts are transported into the cytoplasm, their m6A sites are recognized by a series of m6A readers, such as YTHDF1/2/3, IGF2BP1/2/3 and YTHDC1/2, which then exert regulatory effects [20–24].

New m6A methylation-associated enzymes are frequently identified, and they have been proven to play crucial roles in many essential biological processes, such as cell fate decisions, embryonic development, sex determination, spermatogenesis, tumorigenesis, and T cell homeostasis [25]. In particular, m6A methylation has a wide range of effects on the nervous system and plays important roles in the self-renewal of neural stem cells, learning memory, brain development, and synaptic growth. For instance, specific depletion of m6A in the nervous system by conditional knockout of *Mettl14* causes compromised cortical neurogenesis in embryonic mice by regulating the decay of neurogenesis-related transcripts [26]. Analogously, selective inactivation of METTL3 in the mouse nervous system causes severe developmental defects in the brain by controlling the mRNA stability of genes involved in cerebellar development and apoptosis [27]. In addition, deficiency of the m6A eraser FTO affects neurogenesis and hippocampal-dependent memory in mice [28]. In vitro data further indicate that FTO is enriched in axons, and specifically silencing axonal FTO inhibits axon elongation [29]. Furthermore, deficiency of another m6A eraser, ALKBH5, was reported to affect the RNA metabolism of a subset of cell fate determination genes and thus cause defective postnatal cerebellar development [30]. In addition, *Ythdf1-*deficient mice present diminished learning memory ability with impaired hippocampal synaptic transmission [31]. The proliferation and differentiation of neural stem cells are severely affected by embryonic deletion of *Ythdf2 *[32]. Very recently, the newly found reader Prrc2a was also proven to maintain normal cognition by determining the specification and myelination of oligodendrocytes [33]. All these studies indicate the important roles of epitranscriptomic m6A modifications in the regulation of neuronal development and neurogenesis. However, the in vivo functions and mechanisms of m6A in the neural retina remain to be characterized. Therefore, we focused on exploring the potential role of m6A in retinal photoreceptor cells.

In the present study, we generated the first retina-specific *Mettl14*-knockout mice using the Rho-Cre and HRGP-Cre lines and investigated the functions of *Mettl14* in retinal rod and cone photoreceptors. Our data showed that loss of METTL14 in rod cells causes a weakened scotopic photoresponse and rod degeneration. Further study revealed the ectopic accumulation of multiple OS proteins in the IS. Deficiency of METTL14 in cone cells led to the mislocalization of cone opsin protein and the death of cone cells. Moreover, *Mettl14* depletion resulted in drastic decreases in METTL3/WTAP levels and reduced m6A methylation levels. Mechanistically, transcriptomic analyses in combination with MeRIP-seq illustrated that m6A depletion via inactivation of METTL14 resulted in reduced expression levels of multiple phototransduction- and cilium-associated genes, which subsequently led to reduced levels of OS-resident molecules and compromised ciliogenesis in rod cells. Collectively, our data demonstrated that METTL14 plays an important role in regulating phototransduction and ciliogenesis events and is necessary for photoreceptor function and survival, highlighting the importance of m6A modification in the retina.

## Results

### Establishment of a specific knockout model of *Mettl14* in retinal rod and cone cells

As a key methyltransferase, METTL14 plays important roles in vivo by mediating m6A methylation of mRNA. However, the molecular functions of METTL14 in the retina remain elusive. To determine the role of METTL14 in the retina, we began by examining the expression pattern of *Mettl14* in rod and cone photoreceptors. Western blot data first confirmed that the METTL14 protein was abundant in mouse retinas (Additional file 1: Fig. S1A). Immunofluorescence staining of frozen murine retinal sections revealed that METTL14 was widely expressed in whole retina, and distinctly expressed in the IS and outer nuclear layer (ONL) (Additional file 1: Fig. S1B). In ONL, interestingly, the METTL14 immunostaining was most intense near the nuclear periphery of photoreceptor cells (Additional file 1: Fig. S1B), the region where chromatin appears to be least condensed [34], implying that METTL14 plays a role in m6A modification in photoreceptors.

To investigate METTL14 expression in cone cells, a cone-specific ROSA26-tdTomato reporter line was introduced to monitor the entire cell body of cones (see details in methods), permitting double-labeling analysis with nucleus-localized METTL14. As expected, the immunofluorescence data showed that METTL14 was analogously expressed in the nuclear periphery of tdTomato-expressing cones (Additional file 1: Fig. S1C). Thus, the intense expression of METTL14 in both rod and cone photoreceptors suggests that METTL14 may be involved in the maintenance of photoreceptor cells.

To investigate the potential functional roles of METTL14 in these cells, we generated mice with rod cell-specific METTL14 ablation by crossing *Mettl14* [exons 2 to 10] floxed mice (*Mettl14*^*flox/flox*^) with a Rho-Cre driver line in which Cre is targeted to the *Rho* locus; thus, Cre expression is under the control of the endogenous rod *Rho* promoter [35] (Additional file 1: Fig. S1D). In addition, a METTL14 cone knockout line was constructed using HRGP-Cre mice in which Cre is driven by the human red/green pigment gene promoter [36] (Additional file 1: Fig. S1D). Genotyping results were used to distinguish the littermates of the progeny: *Mettl14*^*flox/flox*^*; Rho-Cre* (hereafter RKO), *Mettl14*^*flox/flox*^*; HRGP-Cre* (hereafter HKO), and *Mettl14*^*flox/flox*^ (used as control, hereafter Ctrl) (Additional file 1: Fig. S1E).

### Specific depletion of *Mettl14* in retinal rods causes impaired visual function

**Fig. 1:**
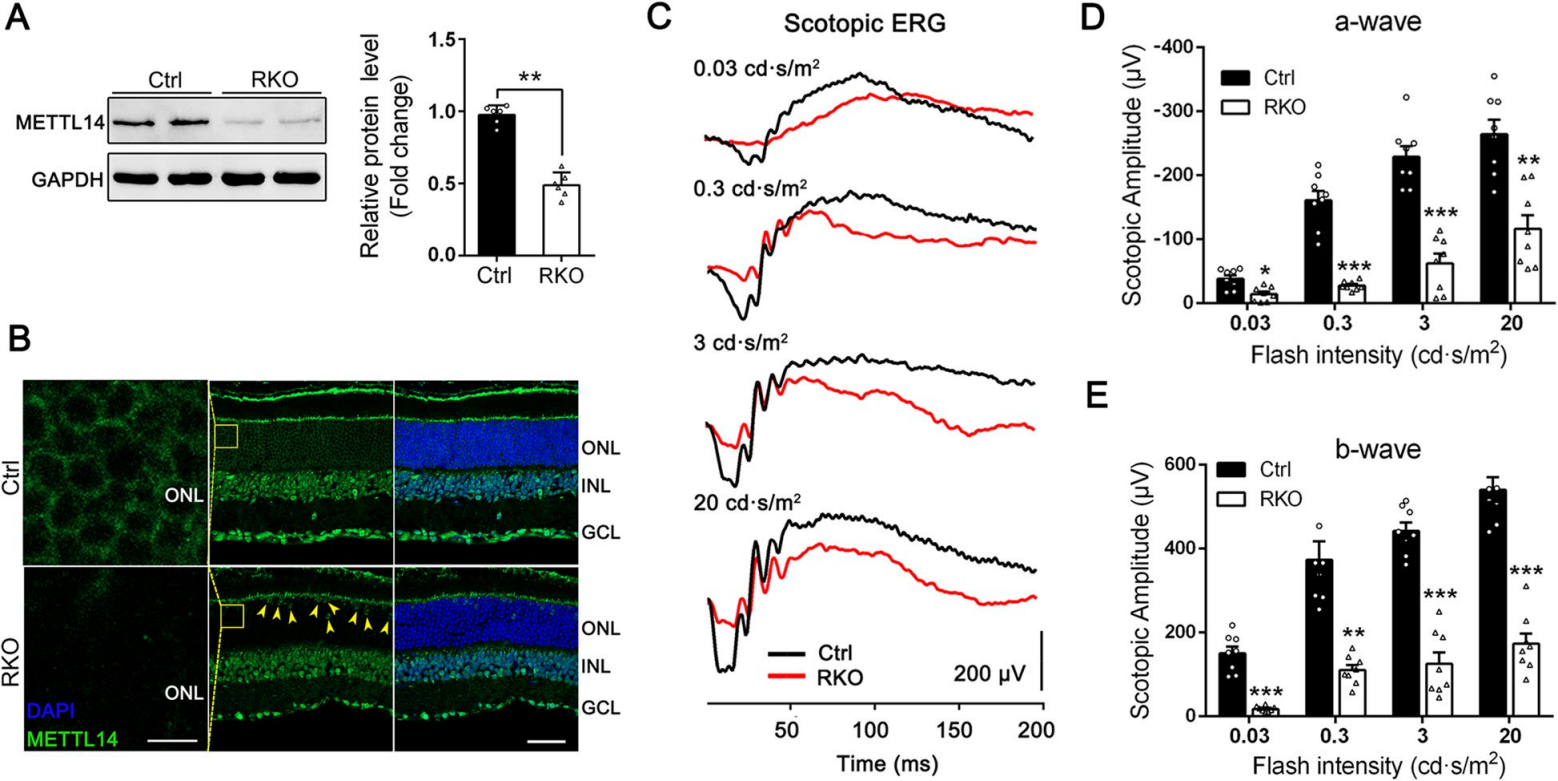
Specific depletion of *Mettl14* in retinal rods led to impaired visual function. **A** Immunoblotting and quantitative comparison of METTL14 protein expression in retinas from *Mettl14*^*flox/flox*^ (Ctrl) and *Mettl14*^*flox/flox*^*; Rho-Cre* (RKO) mice (*n* = 6). GAPDH was used as a loading control. **B** Immunofluorescence labeling of retinal cryosections from Ctrl and RKO mice at the age of 2 months using a METTL14 antibody (green). DAPI (blue) was used to counterstain the nuclei. Arrowheads indicated that METTL14 is still expressed in cone cells in RKO retinas. Scale bar: 25 μm. **C** Representative electroretinogram (ERG) traces corresponding to responses elicited by scotopic conditions at flash intensities of 0.03, 0.3, 3, and 20 cd·sec/m.^2^ in mice at 5 months of age. **D**, **E** Statistical analysis was performed for the amplitudes of the a-wave (**D**) and b-wave (**E**) under scotopic conditions (*n* = 8, two-way ANOVA followed by Tukey’s post hoc test). ONL, outer nuclear layer; INL, inner nuclear layer; GCL, ganglion cell layer. *p < 0.05; **p < 0.01; ***p < 0.001. #, no significant difference. Data are presented as the mean ± SEM

To define the retinal phenotype of the constructed RKO mice, we first confirmed the excision efficiency in rods. Western blot analysis indicated that the METTL14 protein level in the retina in RKO mice was reduced to 53% of that in their control littermates (Fig. 1A). Considering that rod cells account for 97% of photoreceptors but ~ 70% of all retinal cells [37], the deletion efficiency was fairly robust. Immunostaining results further verified that the RKO mice failed to produce the METTL14 protein in the retinal ONL, although METTL14 was still expressed in cones (Fig. 1B).

We next evaluated the physiological status of the RKO retina. Photopic and scotopic electroretinograms (ERGs) of control and RKO mice at 5 months of age were performed. Scotopic ERG recordings, which detect the responses of rod-driven circuits, revealed reduced amplitudes of both a-waves and b-waves compared with those of the controls at each flash intensity (Fig. 1C). The mean amplitudes of the a- and b-waves of RKO mice were reduced by approximately 73% and 67%, respectively (Fig. 1D, E). In contrast, photopic ERG traces, which reflect the status of cone cells, appeared to be normal in RKO mice (Additional file 1: Fig. S2). These results suggested that the rod photoreception function is significantly compromised by the loss of METTL14.

### Rod cell degeneration and mislocalization of OS proteins in the RKO retina

**Fig. 2:**
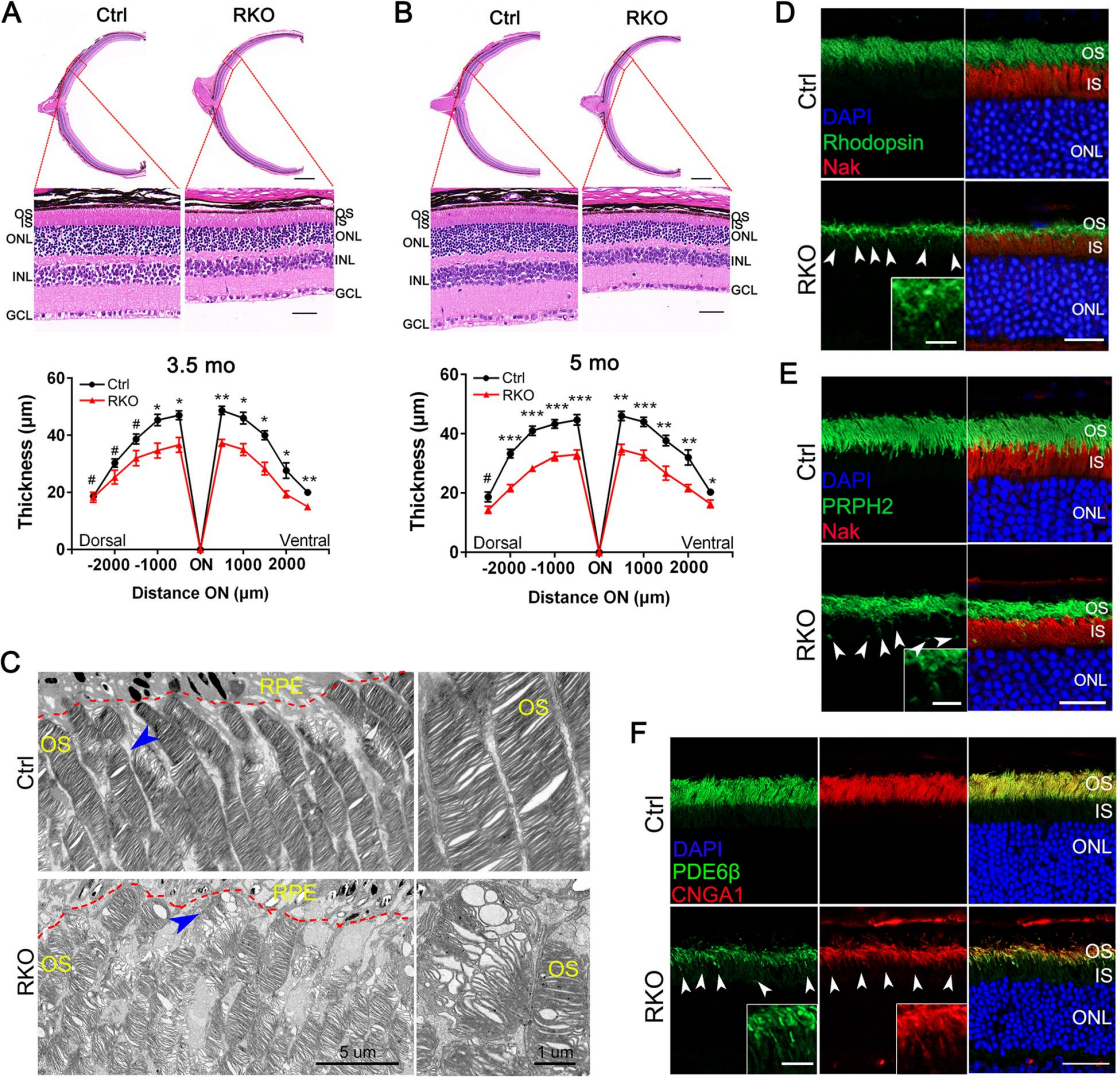
*Mettl14* deficiency led to progressive death of rod cells and mislocalization of OS proteins. **A**, **B** H&E staining of paraffin sections of RKO and corresponding control retinas at the ages of 3.5 (**A**) and 5 months (**B**). Scale bar: 25 μm. The lower panel shows the quantitative analysis of the ONL thickness of the Ctrl (*n* = 8) and RKO (*n* = 6) retinas from mice at different ages. Two-way ANOVA was used for statistical analysis, followed by Tukey’s post hoc test. **C** Representative transmission electron microscopy (TEM) images of photoreceptor outer segments in 3.5-month-old control and RKO mice. Scale bar: 5 μm. Severely disorganized outer segment discs were observed in RKO photoreceptor cells. Arrowheads indicate the outer segments, and enlarged images of outer segment discs are shown in the right panel. Scale bars: 1 μm. **D**, **E** Retinal cryosections from 3.5-month-old mice were labeled with rhodopsin, PRPH2 and the IS marker Na–K ATPase (red). DAPI was used to counterstain the nuclei. Scale bars, 25 μm. The white arrowheads indicate mislocalized OS proteins in the IS. Inset images show a cropped and zoomed image. Scale bars, 1 μm. **F** Representative immunofluorescence images of PDE6B (green) and CNGA1 (red) in retinas from 3.5-month-old Ctrl and RKO mice. Nuclei were counterstained with DAPI (blue). Scale bars, 25 μm. The white arrowheads indicate mislocalized OS proteins in the IS. Inset images show a cropped and zoomed image. Scale bars, 1 μm. OS, outer segment; IS, inner segment; ONL, outer nuclear layer; INL, inner nuclear layer; GCL, ganglion cell layer; RPE, retinal pigment epithelium. *p < 0.05; **p < 0.01; ***p < 0.001. #, no significant difference. Data are presented as the mean ± SEM

To determine the pathological changes underlying the abnormal ERG results, histologic analysis was performed to examine the retinal morphology in RKO retinas at different ages. At 2 months, no obvious difference was observed between the RKO and control mice (Additional file 1: Fig. S3). However, at 3.5 months of age, a shorter OS and thinning of the outer retina were observed in the RKO retina (Fig. 2A). This phenotype worsened at 5 months of age, and the ONL was much thinner, with a 36.6% decrease in thickness (Fig. 2B). At 11-month-old, surprisingly, no OS was left, and the ONL was reduced to 1–2 cell per row in RKO retina (Additional file 1: Fig. S4A). Only trace tissues remained in ONL in 12-month-old RKO mice (Additional file 1: Fig. S4B). In addition, heterozygous *Mettl14*^*loxP/*+^, *Rho-Cre* mice were tested. The heterozygous RKO mice showed a mild degeneration phenotype characterized by a slightly thinner ONL thickness at 9 months of age, while the ONL of their RKO littermates only had 2–3 cell per row at this stage (Additional file 1: Fig. S5). We thus focused on homozygous RKO mice in the following analysis.

To characterize the ultrastructural features of the photoreceptor OS, electron microscopy was performed and revealed severely disorganized outer segment discs of 3.5-month-old animals. In control siblings, photoreceptor OSs were elongated and contained well organized stacks of discs, generating a palisade pattern on the retinal pigment epithelium (RPE). The OS discs, however, were severely disorganized and fragmented in RKO photoreceptor cells (Fig. 2C), indicating the degeneration and compromised maintenance of the OS.

The OS houses large amounts of phototransduction proteins, and all OS proteins are synthesized in the IS before being transported to the OS across the photoreceptor cilium. To investigate the molecular mechanisms underlying these severe phenotypes, we examined the subcellular localizations of multiple key OS proteins in RKO retinas by immunofluorescence staining. Rhodopsin, the key light receptor in rods that initiates scotopic vision, constitutes over 80% of protein in the disk membrane in the OS of rod photoreceptors [38]. At 3.5 months of age, rhodopsin was well organized and strictly restricted to the rod OSs of control animals, and it was absent in the NaK pump antibody-labeled IS (Fig. 2D). In RKO mutants, however, reduced rhodopsin content was observed, together with punctate structures in which rhodopsin was mislocalized to the IS (Fig. 2D). Analogously, we found that the distribution of the OS structural protein peripherin 2 (PRPH2) displayed similar ectopic IS accumulation in RKO retinas (Fig. 2E). Furthermore, the normal localization of both phosphodiesterase-6-β (PDE6B) and CNGA1, the central phototransduction components, was in the OS in the control retina. In contrast, in the RKO retina, increased PDE6B and CNGA1 immunoreactivity was detected in the IS (Fig. 2F). These results demonstrate that METTL14 deficiency impairs the protein transport of several integral phototransduction pathway proteins in rod photoreceptors, which likely contributes to photoreceptor dysfunction and retinal degeneration.

In addition, immunostaining of retinal sections revealed that the expression of glial fibrillary acidic protein (GFAP) was dramatically upregulated in degenerating retinas compared to control retinas (Additional file 1: Fig. S6A). Western blot analysis further confirmed this result, as revealed by the increased GFAP protein level in the RKO retina (Additional file 1: Fig. S6B, C). These results suggested that Müller cells were activated to proliferate as an indicator of retinal damage [39]. TUNEL analysis revealed several TUNEL-positive nuclei in the ONL of retinas from 5-month-old RKO mice (Additional file 1: Fig. S6D, E), suggesting that the gradual loss of photoreceptors in the RKO retina mainly occurs via apoptosis. Together, these data indicate that ablation of METTL14 results in rod photoreceptor degeneration.

### METTL14 depletion in cone cells leads to cone photoreceptor defects

**Fig. 3:**
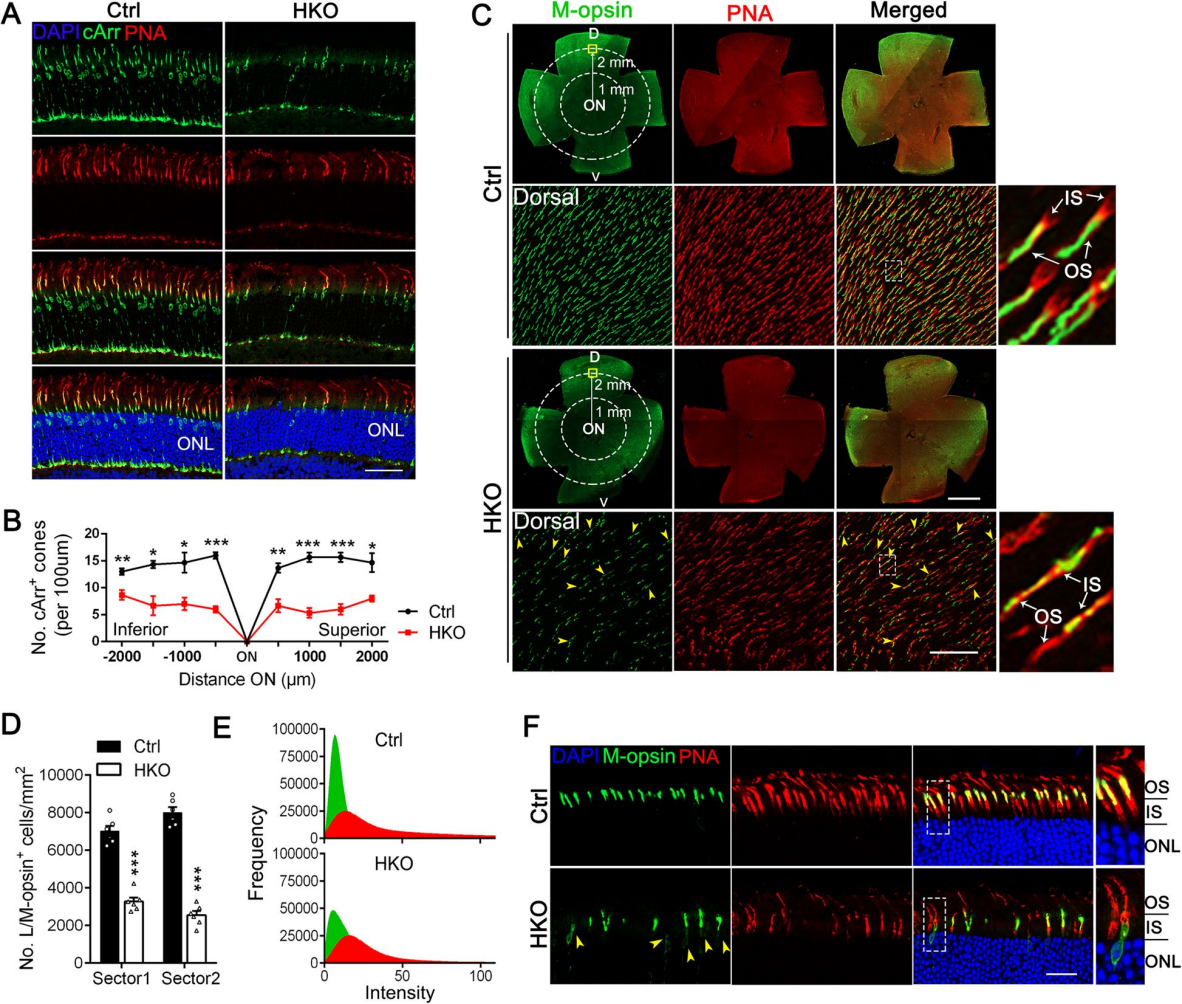
METTL14 depletion in cone cells leads to cone photoreceptor defects. **A** Representative immunofluorescence images of cone arrestin (green) and PNA (red) in retinas from 6-month-old Ctrl and HKO mice. Nuclei were counterstained with DAPI (blue). Scale bars, 25 μm. **B** Number of cone arrestin-marked cones per 500 μm field in Ctrl and HKO retinas (*n* = 6, two-way ANOVA followed by Tukey’s post hoc test). Each cone population was counted in the inferior and superior retinal quadrant starting -2500 μm from the ora serrata and moving toward the optic disc every 500 μm. **C** Immunostaining of flat-mount retinas from 5-month-old control and HKO mice for M-opsin and PNA markers revealed cone cell disorganization in HKO retinas. Scale bars, 50 μm. Representative images from the dorsal retinal quadrant are shown in the lower panel. Yellow arrowheads indicate mislocalized M-opsin in the IS. Inset images show a cropped and zoomed image. Scale bars, 1 μm. Schematic of a retinal flat mount indicating the two sectors (radii: 1 and 2 mm) that were used to count cones. **D** Number of M-opsin-marked cones per 1 mm^2^ field in the two sectors of Ctrl and HKO retinas (*n* = 6). **E** Distribution of the fluorescence intensity of M-opsin (green) and PNA (red) in retinal flat mounts from control and HKO mice. **F** Retinal cryosections from 5-month-old mice were labeled with the cone markers PNA and M-opsin. DAPI was used to counterstain the nuclei. scale bars, 25 μm. Yellow arrowheads indicate mislocalized M-opsin in the IS. The right panels show high-magnification images of the outline areas. Scale bars: 1 μm. OS, outer segment; IS, inner segment; ONL, outer nuclear layer; *p < 0.05; **p < 0.01; ***p < 0.001. #, no significant difference. Data are presented as the mean ± SEM

Cone cells are responsible for daylight, central, high-acuity and color vision. We next determined the effect of METTL14 deficiency on cone cells using the constructed HKO mice. METTL14 depletion in cone cells of HKO animals was confirmed. Given that cones constitute only ~ 3% of retinal photoreceptor cells [34] and using western blotting to confirm the deletion efficiency should be problematic, we introduced ROSA26-tdTomato reporter mice to visualize the specific expression of HGRP-Cre in cone photoreceptors (see details in the Methods). Cone cells were labeled with an antibody specific to cone arrestin (cArr), and HRGP-Cre was distinctly expressed in cArr-marked cone cells (Additional file 1: Fig. S7A), suggesting precise excision. Immunofluorescence staining further verified specific METTL14 depletion in cone cells (Additional file 1: Fig. S7B). Photopic ERG analysis revealed decreased ERG responses in 6-month-old HKO mice in comparison to control (Additional file 1: Fig. S8A, B, E), while no significant difference of scotopic a-wave or b-wave was observed between those two groups (Additional file 1: Fig. S8C, D, F), suggesting impaired cone function. We then double-immunolabeled retinal cryosections from 6-month-old control and HKO mice with cArr as well as Alexa Fluor-594-conjugated PNA, which mainly labels the cone matrix sheaths associated with all three types of cones. The immunofluorescence results revealed a dramatic reduction in cone number in HKO mice (Fig. 3A, B), with a shortened, disorganized, and misshapen cone OS compared to that of controls, suggesting that METTL14 depletion results in the degeneration of cone photoreceptors.

To further examine the pathological changes of cones in HKO mice, retinal flat mounts from young (5-month-old) animals were prepared and coimmunostained with an L/M-opsin antibody to mark the cone OS and Alexa Fluor-594-conjugated PNA. Given that the M-opsin-expressing cones were largely concentrated in the dorsal retina, with very few M-opsin-positive cones in the ventral retina [40], we quantified the OS length and the number of M-cones in the dorsal retina of HKO and control mice. As expected, a dramatic decrease in the number of M-cone cells was observed in the HKO retina (Fig. 3C, D), in line with the immunolabeling results of cArr. In addition, although the M-opsin-marked OS length was comparable across the different groups during this time, the overall M-opsin fluorescence intensity in HKO retinas was significantly decreased (Fig. 3E), suggesting opsin degradation. Notably, close examination of retina cross-sections in higher-magnification images further revealed the extensive accumulation of M-opsin in the IS and cell body, accompanied by a malformed OS (Fig. 3F). Considering the similar mislocalization pattern of OS proteins observed in RKO rods (Fig. 2E-G), we suggest that METTL14 deficiency-induced defects in protein transport to the OS underlie the degeneration of retinal photoreceptors.

### METTL14 deletion decreases the stability of m6A methyltransferases and the abundance of m6A methylation in the RKO retina

**Fig. 4:**
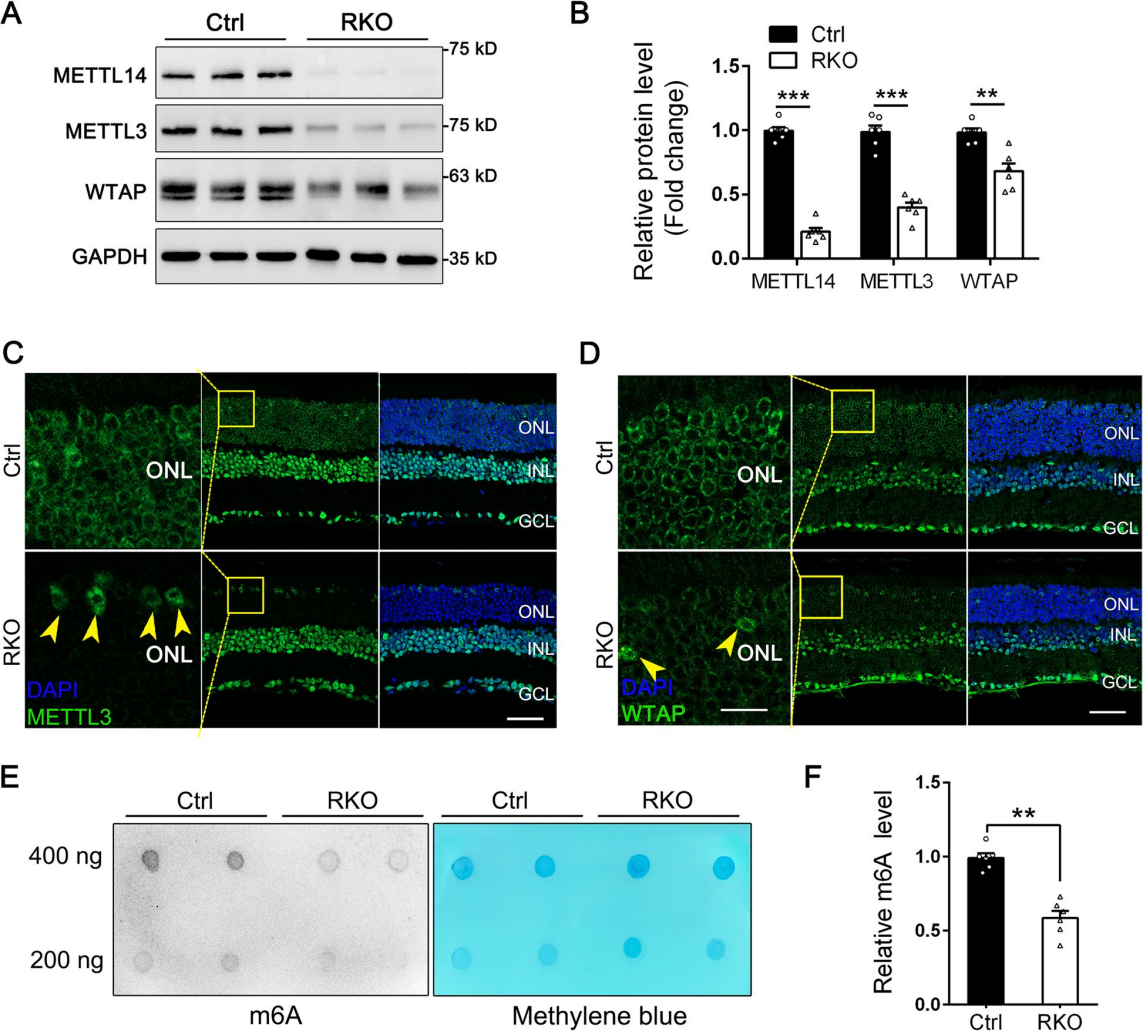
Loss of *Mettl14* led to decreased METTL3/WTAP expression and diminished m6A-RNA methylation in RKO retinas. **A** Reduced expression levels of METTL3 and WTAP in the retinas of 5-month-old RKO mice, as shown by immunoblotting of total lysates of the retinas of Ctrl and RKO mice. **B** Quantification of relative protein levels in Ctrl and RKO retinas (*n* = 6). **C**, **D** Immunofluorescence labeling of retinal cryosections from Ctrl and RKO mice at the age of 5 months using a METTL3 (**C**) or WTAP (**D**) antibody (green). DAPI (blue) was used to counterstain the nuclei. Scale bar: 25 μm. Higher-magnification images of the boxed area are shown in the left panel. Arrowheads indicated that the METTL3/WTAP is still expressed in cone cells. Scale bars: 10 μm. **E** mRNAs were isolated from retinas, followed by dot blot analysis with an m6A antibody. Methylene blue staining was used as a loading control. **F** Quantification of the relative m6A levels in E (*n* = 6). ONL, outer nuclear layer. **p < 0.01; ***p < 0.001. #, no significant difference. Data are presented as the mean ± SEM

METTL14 is known to physically and functionally interact with the RNA methyltransferase METTL3 and the RNA binding protein WTAP to constitute the core components of the RNA methyltransferase complex [41]. In addition, METTL14 and METTL3 were reported to regulate each other’s stability at the protein level [42]. These results prompted us to examine the expression level and cellular distribution of METLL3 and WTAP in METTL14-deficient RKO retinas. Western blot data showed that the expression level of METTL3 was reduced to 37% of that of controls, while the WTAP protein level in the RKO retina was reduced to 68% of that of controls (Fig. 4A, B). Consistent with this finding, using immunofluorescence microscopy, we found that METTL14 depletion decreased the expression of both METTL3 and WTAP to varying degrees in the ONL of RKO retinas, with METTL3 almost absent in rod cells, while WTAP was still present in the ONL but showed a lower content (Fig. 4C, D). In contrast, the remaining METTL3/WTAP signal was still expressed in cone cells of RKO retinas (Fig. 4C, D, arrows). These findings suggested that METTL14 depletion leads to impaired stability of the m6A methyltransferase complex. To investigate whether the altered protein level affects METTL14-mediated m6A methylation, the global profiles of m6A in RKO retinas were detected by dot blot analysis. As expected, there was a 40 to 50% reduction in overall m6A levels in the RKO retina compared with the control retina (Fig. 4E, F). Taken together, these results suggest that METTL14 is critical for the stability of the m6A methyltransferase complex and m6A methylation in retinal rod cells.

### Transcriptome-wide MeRIP-seq and RNA-seq assays to identify potential targets of METTL14

**Fig. 5:**
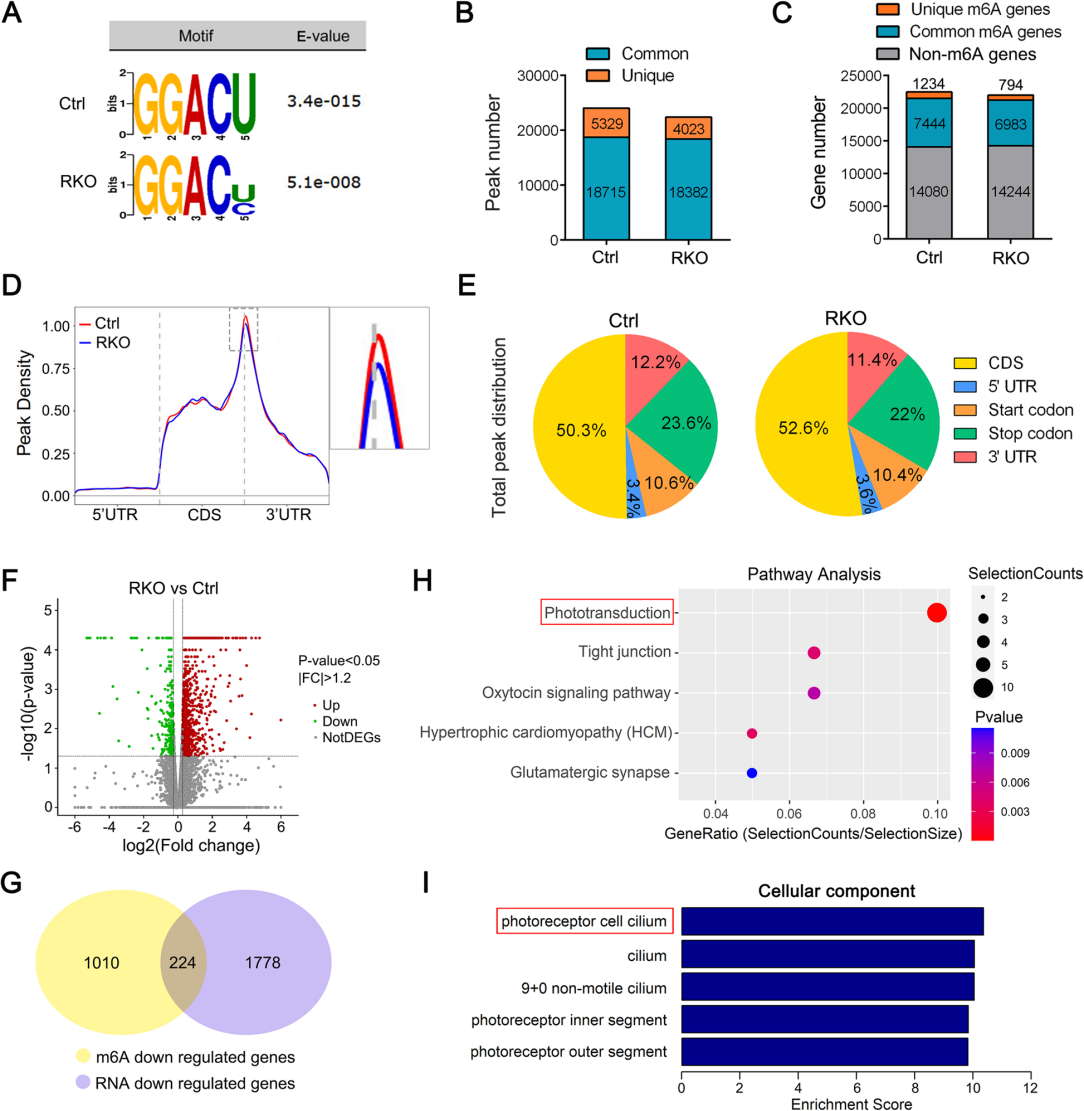
Characterization of m6A modification and gene expression changes in RKO retinas. **A** Top consensus motif identified via HOMER with m6A-seq in Ctrl and RKO retinas. **B** Number of m6A peaks identified by m6A-seq in Ctrl and RKO retinas. **C** Number of m6A-modified genes identified by m6A-seq. Common m6A genes contain at least 1 common m6A peak, while unique m6A genes contain no common m6A peaks. **D** Distribution of m6A peaks in transcript segments divided into the 5′ UTR, CDS, and 3′ UTR in Ctrl and RKO retinas. Enlarged images of the boxed area are shown in the right panel. **E** Graphs of the m6A peak distribution showing the proportion of total m6A peaks in the indicated regions across the entire set of mRNA transcripts. **F** Volcano plots displaying the mRNAs that were differentially expressed between the RKO and Ctrl groups and their statistical significance (fold changes ≥ 1.2 and *p* < 0.05). **G** Venn diagram showing the numbers of shared genes with reduced m6A modification (m6A downregulation) identified by m6A-seq among genes with downregulated expression identified by RNA-seq. **H**, **I** Pathway analysis (**H**) and significantly enriched (*p*-value < 0.01, correction with Benjamini–Hochberg multiple testing) GO terms in cellular component (**I**) categories for 224 genes selected in Fig. 5G

To investigate the variations in m6A modification of specific genes, we performed methylated RNA immunoprecipitation sequencing (MeRIP-seq) on 3-month-old control and RKO retinas, with independent biological replicates. De novo motif analysis using HOMER identified “GGACU” as the most common m6A motif in m6A-immunoprecipitated RNAs from control retinas, while the m6A peaks in RNAs from RKO retinas were enriched in the regions with GGACR motifs (Fig. 5A), in accordance with observations in the mammalian system [20, 43].

In total, m6A-seq identified 24,044 and 22,405 m6A peaks from 8402 and 7777 m6A-modified transcripts in control and RKO retinas, respectively (Fig. 5B). In the RKO retina, 5329 peaks disappeared and 4023 peaks appeared, while the remaining 18,382 peaks were found in both RKO and control retinas (Fig. 5B). Regarding m6A-modified genes, m6A-seq identified 794 newly modified genes and 1234 genes showing loss of m6A modification in the RKO retina, while the other 6983 genes were found in both control and RKO retinas (Fig. 5C). Given that METTL14 is a key component of the m6A methyltransferase complex, these data indicated that the identified unique peaks and genes are supposed to contain genuine targets of METTL14. We further analyzed the density distribution of m6A peaks across the mRNA transcripts, and they were found to be predominantly localized in the stop codon, covering the CDS and 3′ UTR in both control and RKO retinas, although fewer m6A peaks were enriched near the stop codons in the RKO retina (Fig. 5D, E).

To explore how METTL14 depletion affects m6A-containing transcripts, we next employed RNA sequencing (RNA-seq) to identify the affected genes in control and RKO retinas. Considering that rods occupy only the outermost layers of the neurosensory retina, genes with fold change (FC) > 1.2 and adjusted *p*-value < 0.05 were defined as differentially expressed genes. Through analysis of our RNA-seq data, we demonstrated that the mRNA transcript levels of 932 genes were upregulated and those of 2002 genes were downregulated in RKO retinas compared to control retinas (Fig. 5F). We filtered these 2,002 downregulated genes that were found by RNA-seq with 1,234 genes that lost their m6A peaks in the RKO retinas, which resulted in the identification of 224 candidate genes. We next focused on these 224 genes to obtain insights into the molecular mechanisms (Fig. 5G). Gene ontology (GO) pathway analysis of these 224 genes indicated that a set of genes were particularly associated with phototransduction (Fig. 5H). Moreover, analysis of cellular components further revealed that m6A modifications were enriched in a handful of genes related to the photoreceptor cilium (Fig. 5I). All enriched GO terms in terms of biological processes and cellular component are shown in Additional file 2 and 3, respectively. Interestingly, the phototransduction cascade initiates visual processing in the photoreceptor OS, which is actually derived from a highly specialized primary cilium termed the connecting cilium. Ciliary dysfunction generally causes compromised visual processing, which in turn induces degeneration of mature photoreceptors. Therefore, we focused on the phototransduction process and the photoreceptor cilium in the following investigation.

### METTL14 regulates the m6A modification and translation of multiple phototransduction-related genes in rods

**Fig. 6:**
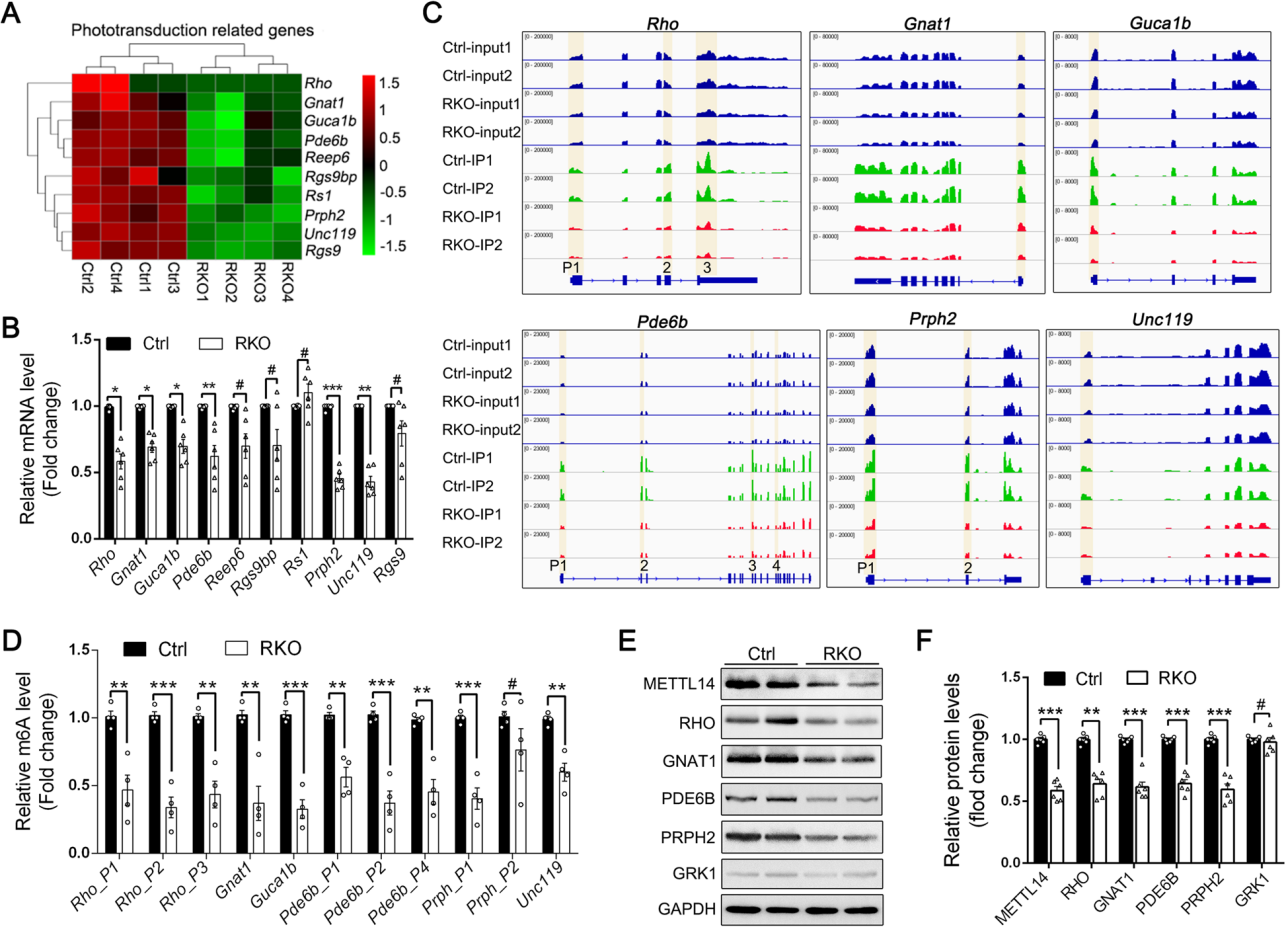
METTL14 mediates m6A modification and controls the expression of multiple phototransduction-related genes. **A** Heatmap of RNA-seq analysis showing that the levels of several phototransduction-related genes were downregulated in RKO retinas. **B** RT-qPCR verified the RNA-seq results for the indicated genes (*n* = 6). **C** Integrative Genomics Viewer (IGV) tracks displaying MeRIP-seq (lower panels, IP) and RNA-seq (upper panels, input) reads along the locus of phototransduction-related genes in Ctrl and RKO retinas. Two replicates are shown. **D** Enrichment of m6A at multiple sites (highlighted in Fig. 6C) in transcripts of significant phototransduction-related genes was determined by MeRIP-qPCR. (E–F) Representative immunoblots (**E**) and quantification (**F**) of significant phototransduction-related protein levels in retinas from 3-month-old mice. Protein expression data were normalized according to GAPDH (*n* = 6). *p < 0.05; **p < 0.01; ***p < 0.001. #, no significant difference. Data are presented as the mean ± SEM

Light absorption and phototransduction by photoreceptors represent the initial steps of vision, and the unveiled phototransduction pathway prompted us to further explore the target genes of METTL14. Heatmaps of the RNA-seq results showed that the mRNA expression of multiple phototransduction-related genes (*Rho*, *Gnat1*, *Guca1b*, *Pde6b*, *Reep6*, *Rgs9bp*, *Rs1*, *Prph2*, *Unc119*, and *Rgs9*) was significantly downregulated in the RKO retina (Fig. 6A). RT-qPCR further confirmed the downregulation of the genes *Rho*, *Gnat1*, *Guca1b*, *Pde6b*, *Prph2*, *and Unc119* in the RKO retina (Fig. 6B).

We then asked whether the altered gene expression caused by METTL14 depletion was a consequence of suppressed m6A methylation. We first compared the m6A peaks of these altered transcripts between control and RKO retinas using the Integrative Genomics Viewer (IGV) tool, which revealed that the m6A sites of the *Rho*, *Gnat1*, *Guca1b*, *Pde6b*, *Prph2*, and *Unc119* transcripts were mainly distributed in start codons, in exons and around stop codons (Fig. 6C, see details in Additional file 4: Table S1). Moreover, METTL14 silencing broadly reduced the m6A methylation abundance in *Rho*, *Gnat1*, *Guca1b*, *Pde6b*, *Prph2*, and *Unc119* in RKO retinas compared to controls (Fig. 6C). Several significantly (FC > 1.5, *p* < 0.05) decreased m6A methylation sites of these transcripts were filtered out and validated by MeRIP-qPCR with specific primers in fragmented mRNA samples (Additional file 4: Table S1), which confirmed the reduced enrichment of m6A at mapped sites in *Rho*, *Gnat1*, *Guca1b*, *Pde6b*, *Prph2*, and *Unc119* (Fig. 6D).

In addition, when we examined the expression of the corresponding proteins by western blotting, we observed a reduction in RHO, GNAT1, PDE6B, and PRPH2 protein levels in the RKO retina (Fig. 6E, F), suggesting that deletion of METTL14 might decrease the m6A levels of these phototransduction-related gene transcripts, ultimately leading to altered expression. Interestingly, however, another key component in phototransduction, GRK1 (which showed no significant differences in RNA or m6A levels in the RKO retina), appeared to have protein levels comparable to those of control littermates (Fig. 6E, F), indicating that METTL14-mediated m6A modification should be selective.

Together, these results demonstrate that a subset of phototransduction-associated genes are downregulated under METTL14-silenced conditions, highlighting the indispensable role of METTL14-mediated m6A modification in rod phototransduction processes.

### m6A depletion suppresses ciliogenesis by reducing the expression of ciliu-associated genes

**Fig. 7:**
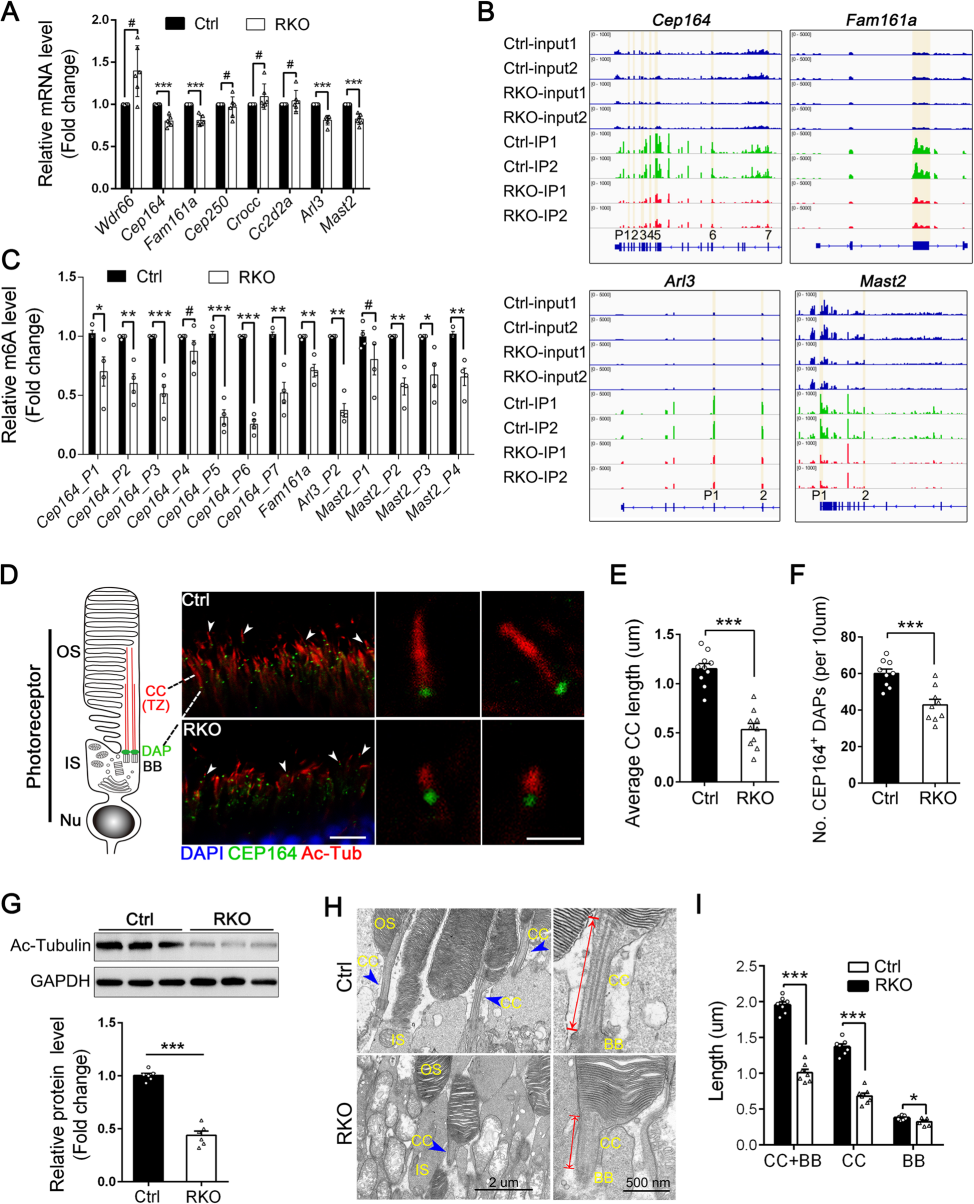
m6A depletion reduces the expression of cilium-associated genes and affects ciliogenesis in rods. **A** RT-qPCR verified the RNA-seq results for cilium-associated genes (*n* = 6). **B** IGV tracks displaying MeRIP-seq (lower panels, IP) and RNA-seq (upper panels, input) reads along the locus of cilium-associated genes in Ctrl and RKO retinas. Two replicates are shown. **C** Enrichment of m6A at multiple sites (highlighted in Fig. 7B) in transcripts of significant cilium-associated genes was determined by MeRIP-qPCR. **D** The left panel shows a schematic diagram of a rod photoreceptor with its ciliary region connecting the inner (IS) and outer segment (OS) compartments, which mainly consists of the connecting cilium (CC) and basal body (BB). The right panel shows representative immunofluorescence images with arrowheads pointing to the ciliary marker acetylated α-tubulin (red) and the distal appendage (DAP) marker CEP164 (green) in retinas from 3-month-old Ctrl and RKO mice. Nuclei were counterstained with DAPI (blue). Scale bars, 5 μm. Higher-magnification images of representative ciliary regions are shown on the right. Scale bars: 1 μm. **E** Quantitative analyses of the length of the CC in Ctrl and RKO photoreceptors (*n* = 10). **F** Quantitation of CEP164-marked DAPs in Ctrl and RKO photoreceptors (*n* = 9). **G** Representative immunoblots and quantification of acetylated α-tubulin protein levels in retinas from 3-month-old mice. Protein expression data were normalized according to GAPDH (*n* = 6). **H** Representative electron micrographs of the ciliary region of photoreceptor cells from 3-month-old Ctrl and RKO mice. Scale bars: 2 μm. Arrowheads indicated typical structure of CC. Higher-magnification images of representative ciliary regions are shown on the right. Scale bars: 500 nm. **I** Quantitative analyses of the length of the BB and CC in Ctrl and RKO photoreceptors (*n* = 7). *p < 0.05; **p < 0.01; ***p < 0.001. #, no significant difference. Data are presented as the mean ± SEM

Given that the genes that encode components of the cilium were also highly enriched, we next explored the roles of METTL14 and its corresponding m6A modification on the photoreceptor cilium. As mentioned above, a subset of downregulated cilium-associated genes with m6A loss were unveiled in the RKO retina, including *Cep164*, *Fam161a*, *Cep250*, *Crocc*, *Cc2d2a*, *Arl3*, and *Mast2*. To validate the sequencing results, we first performed RT-qPCR, and our results showed that *Cep164*, *Fam161a*, *Arl3* and *Mast2* were significantly downregulated in METTL14 RKO retinas (Fig. 7A).

Subsequently, to investigate the impact of METTL14 depletion on the m6A modification of these filtered genes, the m6A peak distribution was monitored using IGV tools. The mRNAs of the indicated cilium-related genes were found to have multiple m6A peaks near the start codon, stop codon, and deposited broadly across multiple exons (Fig. 7B, see details in Additional file 4: Table S1). To better quantify the m6A abundance of these methylated sites in control and RKO retinas, we performed a MeRIP-qPCR assay and detected significant decreases in the enrichment of *Cep164*, *Fam161a*, *Arl3*, and *Mast2* m6A levels in RKO retinas (Fig. 7C), further confirming that our MeRIP-seq data were robust and accurate.

The above findings suggest that METTL14 depletion could reduce the expression of cilium-associated genes. Indeed, among these indicated genes, CEP164 is a centriole DAP protein required for ciliogenesis [44, 45]. FAM161A and ARL3, likewise, were proven to be required for cilium assembly [46–48]. MAST2 is associated with microtubules, although its role in the retina remains unknown [49, 50]. These results remind us that METTL14 presumably maintains the function and survival of photoreceptor cells by regulating the process of cilium formation and assembly. To verify this hypothesis, cryosections of retinas from 3-month-old control and RKO animals were prepared and subjected to immunofluorescence staining with antibodies against the DAP marker CEP164 and the ciliary marker acetylated α-tubulin (Ac-tubulin). Strikingly, photoreceptors in the control retina exhibited elongated and aligned cilia, while RKO photoreceptors presented short, irregular and scattered cilia with a disordered orientation (Fig. 7D). Specifically, the mean cilium lengths were significantly shorter in RKO photoreceptors (Fig. 7E, 1.17 ± 0.08 μm, *n* = 24) than in control photoreceptors (0.53 ± 0.12 μm, *n* = 18), suggesting impaired ciliary assembly. In addition, quantitation of CEP164-marked DAPs revealed a 26.3% decrease in RKO retinas compared to control retinas (Fig. 7F). DAPs, localized in the distal end of the basal body are required for BB docking and subsequent ciliogenesis. Thus, combined with the RNA-seq and RT-qPCR results, it was preliminarily determined that cilium dysfunction in RKO retinas mainly resulted from reduced CEP164 levels and corresponding DAP loss, which ultimately prevented cilium initiation. Consistently, the western blot results further showed that the Ac-tubulin content was clearly attenuated in RKO retinas compared to control retinas (Fig. 7G), confirming the reduced cilium abundance caused by defects in ciliogenesis.

To observe ultrastructural abnormalities, an electron microscope assay was employed and further showed that the absence of METTL14 significantly affected ciliogenesis or cilium assembly in rod cells (Fig. 7H). Although the overall morphology and relative positioning of accessory ciliary structures appeared normal, there was a decrease in the length of the CC and BB by at least 50% compared with that of the age-matched control mice (Fig. 7H, I), consistent with Ac-tubulin immunolabeling. In particular, the CC average length was dramatically decreased in 3-month-old RKO photoreceptors (1.37 ± 0.05 μm) compared with control photoreceptors (0.68 ± 0.06 μm), while the BB in RKO tissues had a slightly reduced length (Fig. 7H, I), suggesting that the BB may indeed fail to undergo complete maturation and subsequent elongation. The CC of photoreceptor cells plays a crucial role in the selective transport of proteins and other molecules to and from the OS. Based on the above mentioned mislocalization of phototransduction proteins in both RKO and HKO retinas, our study demonstrated that *Mettl14* deficiency impairs photoreceptor ciliogenesis by decreasing expression of associated ciliary proteins, which in turn induces ectopic accumulation of OS proteins and ultimately photoreceptor degeneration.

## Discussion

Among posttranscriptional modifications, m6A methylation is the most abundant mammalian internal mRNA modification, and it plays a central role in translational control. However, the necessity of m6A tagging for the normal function of retinal photoreceptor cells has not been definitively demonstrated. In the present study, by using cell type-specific conditional depletion of *Mettl14* in either rod or cone photoreceptors, we show that METTL14-catalyzed m6A modification is indispensable for the function and survival of both rods and cones, likely via the translational regulation of phototransduction- and ciliogenesis-associated genes, which consequently leads to the downregulation and ectopic accumulation of multiple phototransduction molecules residing in the OS and ultimately induces photoreceptor degeneration. Our findings demonstrated, for the first time, the regulatory function of m6A modification in mammalian retinal photoreceptors.

Using high-throughput MeRIP-seq and RNA-seq, we investigated the regulation of METTL14-mediated m6A modification in photoreceptors and revealed two potential mechanisms underlying retinopathy in RKO mice. One potential mechanism involves altered expression of phototransduction-related genes, as numerous key molecules involved in the phototransduction cascade and associated structural OS proteins are modified by m6A, directly affecting the visual impulses of rod photoreceptors. Here, we demonstrated that the transcripts of several phototransduction signaling genes, including the key components in the RHO-Transductin-PDE6 pathway (*Rho*, *Gnat1*, *Pde6b*, *Guca1b*, *Unc119*) and the structural OS protein-encoding gene *Prph2*, present lower levels of m6A methylation (Fig. 6A, B)*.* In addition, a reduction in RHO, GNAT1, PDE6B, and PRPH2 protein levels was detected in the RKO retina (Fig. 6E, F), which likely contributes to the dysfunction of rod cells. It is unlikely that reduced phototransduction protein levels in the RKO retina were the cause of rapid photoreceptor cell degeneration, as GRK1, another key phototransduction protein located in the OS, was present at levels comparable to those of control littermates during this period (Fig. 6E, F), suggesting that the expression changes of these genes are mainly caused by METTL14 depletion-induced m6A loss rather than photoreceptor degeneration. Surprisingly, all of the identified genes play key roles in light transduction, and deletion/mutation of these genes could lead to severe retinal denegation in humans and mice. For instance, mutations in *Rho* are a frequent cause of retinitis pigmentosa (RP) and less often congenital stationary night blindness (CSNB) [51]. The gene *Gnat1*, which encodes the transducin alpha subunit, has also been implicated in CSNB [52] and late-onset rod-cone dystrophy [53]. *Pde6b*, encoding the catalytic beta subunit of PDE6, was one of the first genes identified as causing autosomal-recessive RP and CSNB in humans [54], and *Pde6b* mutant mice (*Pde6b*^*rd1*^ and *Pde6b*^*rd10*^) exhibit rapid photoreceptor degeneration, diminished ERG responses, and abnormal OS morphology [55]. *Guca1b*, which encodes guanylyl cyclase activating protein 2 (GCAP2), a calcium-binding protein that activates photoreceptor GCs, is also associated with RP in humans [56]. In addition, UNC119, which was originally identified in a screen for candidate retinal degeneration genes [57], predominates in the photoreceptor IS and synaptic regions and is essential for transducin trafficking toward the OS [58]. Apart from these, PRPH2 is a photoreceptor-specific glycoprotein necessary for the stabilization and compaction of OS disks, and mutations of PRPH2 have also been associated with RP and macular/pattern dystrophies [59]. PRPH2-deficient (*rds/rds*) mice fail to develop OSs and undergo subsequent retinal degeneration [60]. As stated above, all of these identified genes play vital roles in photoreceptors and are associated with retinopathy. It makes sense that the downregulation of these genes in the RKO retina contributes to photoreceptor dysfunction and degeneration.

In addition, we also filtered out four molecules in the ciliary pathway, *Cep164*, *Fam161a*, *Arl3*, and *Mast2*, which are crucial for the early ciliogenesis steps (BB migration and docking) as well as the subsequent assembly process. Specifically, CEP164, a key component of DAPs, has been proven to be required for BB docking and ciliogenesis. In humans, recessive mutations of CEP164 are associated with nephronophthisis and retinal degeneration [61]. Mice with germline knockouts of CEP164 present defects in BB docking to cell membranes, thereby generating syndromic ciliopathies [62]. Additionally, disruption of CEP164 in RPE cells blocked primary cilium formation [63]. Consistent with these studies, our results indicate a 26.3% decrease in CEP164-marked DAPs in the RKO retina (Fig. 7D, E), which consequently presented fewer and disordered CCs, suggesting dysfunction of ciliogenesis. Another gene identified in this study was *Arl3*, which encodes a protein essential for elongation of tubules; deletion of this gene could prevent formation of an axoneme[48]. In particular, the BBs in *Arl3*^*−/−*^ photoreceptors could dock to the cell membrane without CCs, suggesting that ARL3 does not affect BB docking but prevents the formation of axonemes. Since we detected reduced m6A content and of *Arl3* expression levels in the RKO retina and further showed that the cilium length in RKO retinas was decreased (Fig. 7D, F), it is reasonable to speculate that the absence of *Mettl14* affects not only ciliogenesis but also cilium assembly. In addition, we revealed the downregulation of *Fam161a*, which encodes Fam161a, which is localized to the base of the CC, the BB region, and the adjacent centriole in photoreceptor cells [46]. Mutations in *Fam161a* are the most common cause of autosomal recessive retinitis pigmentosa in the Israeli-Jewish population [64], implying the vital roles of this protein in RP pathologies. Moreover, MAST2, which is a microtubule-associated protein essential for cell division, survival and tumorigenesis, was also identified here [50]. However, until now, little has been known about the role of MAST2 in the retina. Our study suggests that it likely plays a role in photoreceptors, thereby providing clues for further investigation.

Notably, ciliary defects in photoreceptors usually result in aberrant OS protein accumulation and are associated with several forms of inherited retinal degeneration [65]. Analogously, we showed that multiple OS proteins cannot be properly translocated and are mislocalized in the IS in both *Mettl14*-deficient rods (Fig. 2D- F) and cones (Fig. 3F), mainly due to impaired cilia biogenesis. Protein aggregation disrupts photoreceptor function and produces cytotoxic effects, which ultimately accelerate cell death. Indeed, abnormal ciliogenesis and blunted ciliary trafficking are major causes of a wide variety of clinical manifestations commonly referred to as ciliopathies [66], yet the pathogenic mechanisms remain largely unknown. We, for the first time, demonstrated that m6A methylation has a regulatory effect on cilium biogenesis, thereby offering a new viewpoint to elucidate the mechanism of related ciliopathies.

## Conclusions

Our study is the first to demonstrate that m6A is critical for maintaining the function and survival of retinal photoreceptors. We reported that METTL14 deficiency in retinal photoreceptors induces attenuated m6A methylation followed by transcriptome-wide gene expression dysregulation. We provide evidence that METTL14 inhibition suppresses retinal phototransduction and ciliogenesis processes by decreasing the expression of specific phototransduction- and cilium-related genes. Considering the critical role of phototransduction and ciliary pathways in RP retinopathy, this study provides novel insights for RP therapeutic intervention.

## Methods

### Mouse models

All animal experiment protocols were approved by the Institutional Animal Care and Use Committee of Sichuan Provincial People’s Hospital (Chengdu, Sichuan, China) and conducted in accordance with the ARVO Statement for the Use of Animals in Ophthalmic and Vision Research. All animals were housed in a temperature-controlled room (24 °C) with a 12-h light/dark cycle. Mice were maintained on a C57BL/6 J background.

Mice with a *Mettl14* deletion specifically in retinal rod or cone cells were generated using the Cre-loxP system. Briefly, *Mettl14*^*loxP/loxP*^ mice [67] were crossed with *B6.Cg-Pde6b* <  +  > *Tg(Rho-icre)1Ck/Boc* (Jackson Laboratory, stock number: 015850) or *Tg(OPN1LW-cre)4Yzl/J* (Jackson Laboratory, stock number: 0032911) transgenic mice to yield progeny with the genotype *Mettl14*^*loxP/*+^*; Rho/HRGP-Cre*. Then, the *Mettl14*^*loxP/*+^*; Rho/HRGP-Cre* mice were crossed with *Mettl14*^*flox/flox*^ animals to generate *Mettl14*^*flox/flox*^*; Rho -Cre* (RKO) or *Mettl14*^*flox/flox*^*; HRGP -Cre* (HKO) mice. *Mettl14*^*flox/flox*^ littermates were used as controls. In addition, to monitor the expression pattern of the Cre enzyme, a tdTomato reporter gene was used (strain name: *Cg-Gt(ROSA)26Sortm14(CAG-tdTomato)Hze/J*; Jackson Laboratory, stock number: 007914). In the presence of the Cre enzyme, the stop codon before the tdTomato expression cassette was removed, which permits the expression of tdTomato (red fluorescence) in Cre-positive cells.

### Genotyping

Mouse genomic DNA samples were extracted from mouse tails and genotyped using PCR. The floxed *Mettl14* alleles and Rho-Cre or HRGP-Cre were genotyped using the corresponding primers (Additional file 4: Table S2). The tdTomato mice were genotyped using primers provided by the JAX mouse service (Additional file 4: Table S2). All amplification reactions were performed using a master mix (Invitrogen, CA, USA) according to the manufacturer’s instructions. The first cycle of PCR consisted of holding at 95 °C for 5 min, followed by 32 cycles of 95 °C for 15 s, 60 °C for 30 s, and 72 °C for 30 s. The PCR products were separated by DNA electrophoresis on a 3% agarose gel.

### Electroretinography (ERG)

For electroretinographic evaluation of mutants, following overnight dark adaptation, mice were anesthetized with an intraperitoneal injection of xylazine (80 mg/kg) and ketamine (16 mg/kg) in normal saline. Additional anesthetic was given if akinesia was inadequate. The equipment and protocol used here have been described previously [68]. Briefly, the responses of dark-adapted, rod-mediated ERGs to short-wavelength flashes generated by a photopic stimulator over 4.0-log units to the maximum intensity were recorded. Cone-mediated ERGs were recorded in response to white flashes after 10 min of complete light adaptation. The signals were sampled at 0.8 ms intervals and averaged.

### Histological analysis

For hematoxylin and eosin staining (H&E), enucleated eyes from control and mutant mice were fixed overnight in 1.22% glutaraldehyde and 0.8% paraformaldehyde in 0.08 M phosphate buffer, embedded in paraffin and then cut into 5 μm sections. To ensure that sections used for quantification came from the same eccentricity, the globe was embedded in the same orientation. Sections that encompassed the optic nerve (ON) were selected for staining with H&E according to a standard protocol.

### Immunohistochemistry

For immunohistochemistry, eyes were removed from euthanized mice by intraperitoneal injection of pentobarbital (75 mg/kg) and by cervical dislocation and fixed in 4% paraformaldehyde in 100 mM phosphate buffer (pH 7.4) for 1 h at 4 °C, followed by cryoprotection in 30% sucrose for 2 h. The lenses were removed, and the eyes were embedded in optimal cutting temperature (OCT) solution and sectioned at a 10-μm thickness. After blocking and permeabilization with 10% normal donkey serum and 0.2% Triton X-100 in phosphate buffer for 1 h, the sections were labeled with the primary antibody overnight at 4 °C. The primary antibodies used are shown in Additional file 4: Table S3. The sections were rinsed in PBS three times, Alexa Fluor 594/488-conjugated goat anti-mouse/rabbit secondary antibody (Cat# A11005 and A11008, Invitrogen, Waltham, MA, USA, 1:500 dilution) was applied, and nuclei were counterstained with DAPI (Cat# D8417, Sigma, St Louis, MO, USA) for 1 h at room temperature. Images were captured on a Zeiss LSM 800 confocal scanning microscope. In addition, the fluorescence intensity was quantified using ZEN 2.3 imaging software.

### Quantification of cone survival

Retinal whole mounts were prepared as previously described [69]. Briefly, retinas were dissected from enucleated eyes and flattened. The retinas were immersed in 4% PFA for 24 h at 4 °C, blocked in PBS containing 1% bovine serum albumin and 0.5% Triton X-100 and incubated with a polyclonal rabbit anti-L/M-Opsin antibody for 12 h at 4 °C. After several washes, the retinas were incubated with Alexa Fluor 594-conjugated peanut agglutinin (PNA) and an AlexaFluor 488-conjugated goat anti-rabbit secondary antibody (Cat# A11008, Invitrogen, Waltham, MA, USA, 1:250 dilution) for 4 h at room temperature and subsequently washed with PBS. To precisely evaluate the degeneration of cones, the dorsal–ventral orientation of each retina was determined using previously described guidelines [70], and the retina was then subdivided into 2 sectors with radii of 1 mm and 2 mm. Cones were counted manually in the dorsal squares in each sector to determine the average cone density (cones/mm^2^). Details about the antibodies and immunohistochemistry procedure are provided in Additional file 4: Table S3.

### TUNEL assay

Apoptotic cell death was detected by the terminal deoxynucleotidyl transferase-mediated biotinylated UTP nick end labeling (TUNEL) assay according to the manufacturer’s protocol (Cat#11,684,795,910, Roche Diagnostics, Indianapolis, IN, USA) using prepared frozen sections. Images were captured on a Zeiss LSM 800 confocal scanning microscope.

### Transmission electron microscopy (TEM)

Anesthetized mice were fixed by transcardial perfusion with PBS followed by 2.5% glutaraldehyde in cacodylate buffer. The eyes were dissected and postfixed in 2.5% glutaraldehyde in cacodylate buffer, pH 7.2, overnight at 4 °C, and then sectioned at 100-mm intervals using a vibratome. The retinal sections were incubated in 1% osmium tetroxide for 1 h, washed in 0.1 M phosphate buffer, dehydrated via an ascending series of ethanol and propylene oxide, and embedded in Epon (25 g of Epon 812, 13 g of dodecenyl succinic anhydride, 12 g of methyl nadic anhydride, and 1 ml of 2,4,6-tris (dimethylaminomethyl) phenol (DMP-30), Electron Microscopy Sciences). Ultrathin Sects. (70 nm) were cut and stained with uranyl acetate and lead citrate. The sections were imaged under a Philips CM120 scanning transmission electron microscope.

### RNA m6A dot blot assays

mRNA was purified from total RNA using PolyATtract® mRNA Isolation System III (Cat#Z5300, Promega, Madison, WI, USA) following the protocols supplied with the kit. The mRNAs were subjected to doubling dilution with DEPC water and spotted onto a NC membrane (Cat# HATF00010, Millipore, Billerica, MA, USA). The membranes were dried and then UV crosslinked at 1200 W, blocked with 5% skim milk and incubated with an m6A antibody and with anti-mouse or anti-rabbit HRP-conjugated secondary antibodies (1:5000; Bio-Rad, Hercules, CA, USA), and the signal was developed using SuperSignal West Pico Chemiluminescent Substrate according to the manufacturer’s instructions (Pierce, Rockford, IL, USA). The same RNAs were spotted on the membrane and stained with 0.02% methylene blue in 0.3 M sodium acetate as the loading control.

### Methylated RNA immunoprecipitation (MeRIP)

Total RNA was extracted using TRIzol (Cat# Solarbio, Beijing, China). RNA was purified with an EasyPure RNA Purification kit (Cat# ER701-01, TransGen, Beijing, China). The MeRIP assay was carried out with a Magna MeRIP m6A kit (Cat# 17–10,499, Millipore, Billerica, MA, USA) in accordance with the manufacturer’s protocols. m6A modification of particular genes was detected by reverse transcription-quantitative polymerase chain reaction (RT-qPCR) with specific primers (primer sequences are shown in Additional file 4: Table S2).

### RNA-seq and m6A MeRIP-seq

RNA sequencing analysis was performed on four independent biological replicates from control and RKO retinas at 3 months of age. After harvesting, both retinas for each animal were collected and immediately frozen. The samples were sent to Novogene Corporation Inc. (Beijing, China) for high-throughput MeRIP-seq and RNA-seq on an Illumina HiSeq 2500 platform, and 150 bp paired-end reads were generated. All gene expression values from RNA-seq or m6A MeRIP-seq were changed to a log2 value and further analyzed. Then, a *p* value of less than or equal to 0.05 was considered to indicate significance.

### RNA extraction and RT-qPCR

Retinal total RNA was extracted using TRIzol reagent (Cat# T9424, Sigma, Saint Louis, MO, USA) as recommended by the manufacturer. First-strand cDNA was synthesized using an iScript cDNA Synthesis Kit (Cat# 170–8890, Bio-Rad, Hercules, CA, USA). Quantitative PCR was carried out using iTaq SYBRMix (Cat# 1,725,120, Bio-Rad, Hercules, CA, USA) and a CFX384 Touch Real-Time PCR Detection System (Cat# BJ005303, Bio-Rad, Hercules, CA, USA). Primers were designed using Primer3Plus or obtained from published resources. Additional file 4: Table S2 shows the specific primer sequences used. All target genes were normalized to actin mRNA levels, and the fold change was calculated by performing delta-delta Ct analysis.

### Western blotting

Tissues were lysed in radioimmunoprecipitation assay buffer containing a protease inhibitor cocktail (Cat# 11,697,498,001, Roche, Redwood City, CA, USA) and a phosphatase inhibitor (Cat# 4,906,845,001, Roche, Redwood City, CA, USA). The protein concentration of the lysates was determined using a DC Protein Assay according to the manufacturer’s instructions (Cat# 500–0122, Bio-Rad, Hercules, CA, USA). Equal amounts of protein were separated on SDS polyacrylamide gels and transferred onto NC membranes (Cat# HATF00010, Millipore, Billerica, MA, USA). The blots were blocked with 8% nonfat dry milk in Tris-buffered saline (TBS) solution with Tween® 20 detergent for 2 h at room temperature. Then, the membranes were incubated with the primary antibodies in blocking solution overnight at 4 °C. The primary antibodies used for western blotting are shown in Additional file 4: Table S3. The primary antibodies were detected with anti-mouse or anti-rabbit HRP-conjugated secondary antibodies (1:5000; Bio-Rad, Hercules, CA, USA), and the signal was developed using SuperSignal™ West Pico PLUS Chemiluminescent Substrate (Cat# 34,577, Thermo, Waltham, USA). The relative intensity of the immunoreactive bands was quantified using the gel analysis tool provided in ImageJ software. The intensity of the proteins of interest was normalized to that of GAPDH. At least three independent western blots were conducted, and one typical blot is presented.

### Statistical analysis

Statistical analysis was performed using the GraphPad Prism 6 software. The data sets were tested for a normal distribution using the Shapiro–Wilk test. For normally distributed data, statistical significance was determined by Student’s *t*-test or ANOVA. If the data were not normally distributed, a nonparametric statistic was used. *p*-values were calculated with Student’s *t*-test, one-way or two-way ANOVA followed by Tukey’s, Dunnett’s, or Sidak’s multiple comparisons test as appropriate. *p* < 0.05 was considered to indicate statistical significance.

## Supplementary Information

**Additional file 1: Fig. S1.** Generation of the *Mettl14* rod knockout mouse model. **Fig. S2.** RKO mice showed normal photopic ERG response at 5-month-old. **Fig. S3.** 2-month-old RKO mice presented normal retinal structure. **Fig. S4.** Retinal ONL was almost disappeared in aged RKO mice. **Fig. S5.** Heterozygous RKO mice showed retinal degeneration at 9-month-old. **Fig. S6.** Inflammatory response and apoptosis in RKO retinas. **Fig. S7.** Cre-mediated excision of METTL14 in HKO mice. **Fig. S8**. Visual function analysis of HKO mice.

**Additional file 2. **Enriched GO terms in terms of biological processes.

**Additional file 3. **Enriched GO terms in terms of cellular component.

**Additional file 4: Tables S1.** Information of selected differentially methylated RNA sites. **Tables S2.** Primers used in this study. **Tables S3.** Immunological antibodies used in this study.

## Data Availability

The data that supports the findings of this study are available in the supplementary material of this article. Supporting data is included in the Additional files 1, 2, 3, 4. Raw data for m6a sequencing study of *Mettl14* RKO retinas was deposited into SRA (Sequence ReadArchive). The access link is as below: https://www.ncbi.nlm.nih.gov/Traces/study/?acc=PRJNA756938.

## Abbreviations

m6A: N6-methyladenosine
OS: Outer segment
IS: Inner segment
ONL: Outer nuclear layer
INL: Inner nuclear layer
GCL: Ganglion cell layer
CC: Connecting cilium
TZ: Transition zone
BB: Basal body
DAPs: Distal appendages
IRD: Inherited retinal dystrophy
RP: Retinitis pigmentosa
CSNB: Congenital stationary night blindness
Ctrl: Control
WT: Wildtype
RKO: Rho-Cre mediated knockout
HKO: HRGP-Cre mediated knockout
MeRIP: Methylated RNA immunoprecipitation
TUNEL: Terminal deoxynucleotidyl transferase-mediated biotinylated UTP nick end labelling
TEM: Transmission electron microscopy
H&E: Hematoxylin and eosin
OCT: Optimal cutting temperature solution
RT-qPCR: Reverse transcription-quantitative polymerase chain reaction
ERG: Electroretinography
GO: Gene ontology
IGV: Integrative Genomics Viewer
FC: Fold change
