# Supplementary material for: Mettl14-mediated m6A modification is essential for visual function and retinal photoreceptor survival
Source: BMC Biol. 2022 Jun 13;20:140. doi: 10.1186/s12915-022-01335-x (PMC9195452; doi:10.1186/s12915-022-01335-x)
Supplement: Supplementary file 1 — Additional file 1: Fig. S1. Generation of the Mettl14 rod knockout mouse model. Fig. S2. RKO mice showed normal photopic ERG response at 5-month-old. Fig. S3. 2-month-old RKO mice presented normal retinal structure. Fig. S4. Retinal ONL was almost disappeared in aged RKO mice. Fig. S5. Heterozygous RKO mice showed retinal degeneration at 9-month-old. Fig. S6. Inflammatory response and apoptosis in RKO retinas. Fig. S7. Cre-mediated excision of METTL14 in HKO mice. Fig. S8. Visual function analysis of HKO mice. [file 12915_2022_1335_MOESM1_ESM.pdf]

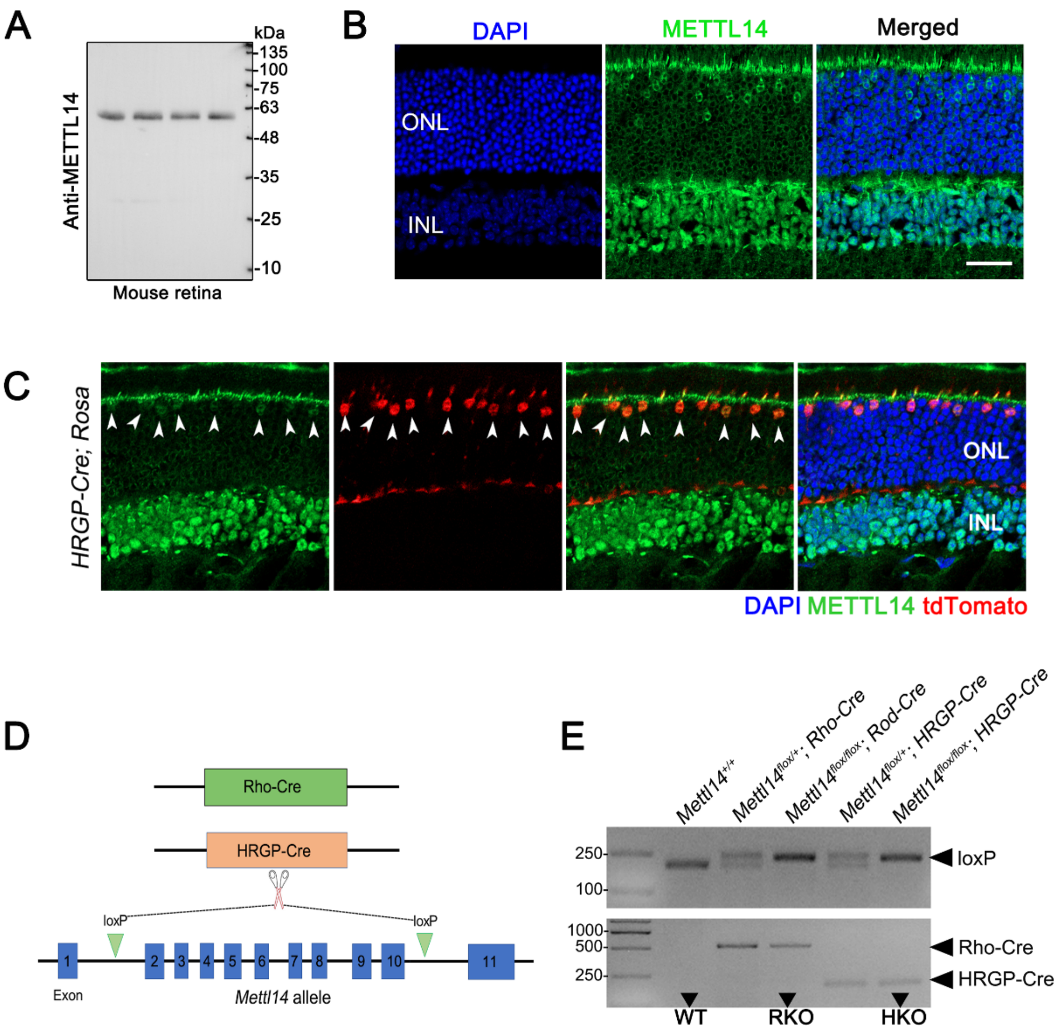

Fig. S1. Generation of the *Mettl14* rod knockout mouse model.

(A) Immunoblotting of METTL14 protein expression in retinas from 2-month-old wild-type (WT) mice. (B) Immunofluorescence staining of cryosections of retinas from WT mice at 2 months of age using METTL14 (green) antibody. Nuclei were counter stained with DAPI (blue). Scale bar: 25 µm. (C) The ROSA-tdTomato reporter was introduced into the mice to monitor HRGP-Cre expression in cones. The retinal section was labeled with METTL14 antibody (green). Nuclei were counter-stained with DAPI (blue). Arrowheads indicated that METTL14 were distinctly expressed in the tdTomato-expressing cone cells (red). (D) Schematic showing the strategy for generation of *Mettl14*$^{flox/flox}$*; Rho-Cre* (RKO) and *Mettl14*$^{flox/flox}$*; HRGP-Cre* (HKO)mice. In the *Mettl14* conditional knockout allele, *Exon 2-10* is flanked by two loxP sites. When the floxed allele is crossed with the Rho-Cre/HRGP-Cre expressing line, *exon 2-10* is deleted, resulting in a frame-shifting deletion allele and

disruption of *Mettl14* gene expression in rod/cone cells. (E) Genotyping of RKO and HKO mice. Genomic DNA from mouse-tail lysate of WT, RKO, and HKO mice were amplified using primer pair *Mettl14*-loxP-F and *Mettl14*-loxP-R, as well as the Rho-Cre-F and Rho-Cre-R or HRGP-Cre-F and HRGP-Cre-R.

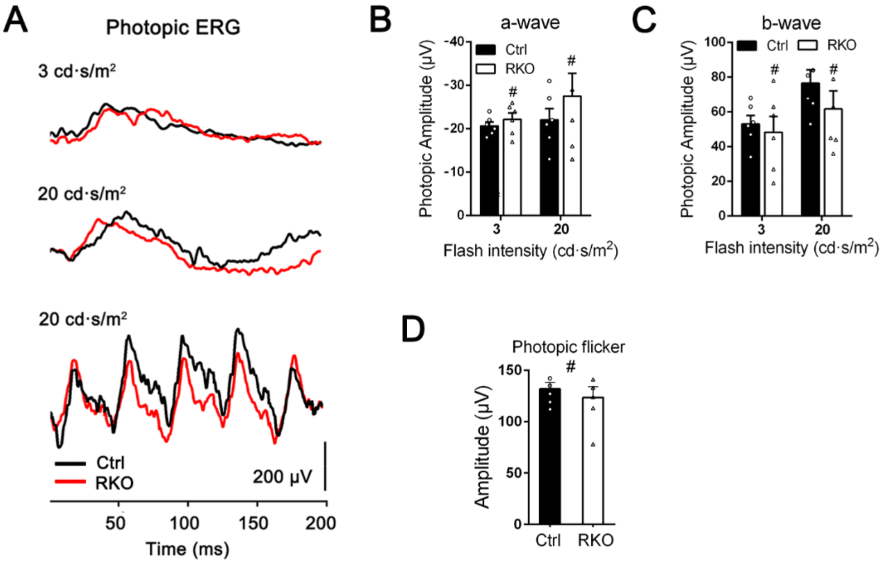

Fig. S2. RKO mice showed normal photopic ERG response at 5-month-old.

(A) Representative electroretinogram (ERG) traces corresponding to responses elicited by photopic conditions at flash intensities of 3 and 20 cd sec/m2 in mice at 5 months of age. (B-D) Statistical analysis were performed for the amplitudes of a-wave (B), b-wave(C) and flicker (D) in photopic conditions (n=8). #, no significant difference. The data are represented as means ± SEM.

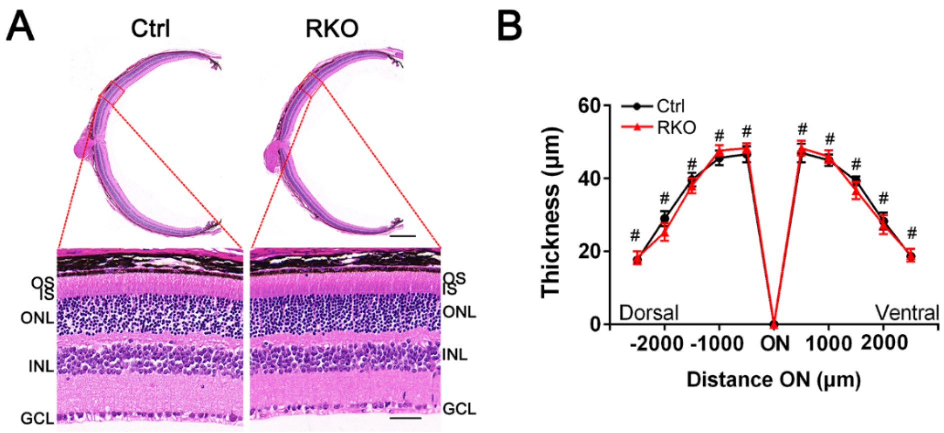

Fig. S3. 2-month-old RKO mice presented normal retinal structure.

H&E staining of paraffin sections of the RKO and corresponding control (Ctrl) retinas at the ages of 2 months. Scar bar: 25 μm. Right panel indicates the quantification analysis of the ONL thickness of the Ctrl (n=8) and RKO (n=6) retinas from mice at 2-month-old. OS, outer segment; IS, inner segment; ONL, outer nuclear layer; INL, inner nuclear layer; GCL, ganglion cell layer. Two-way ANOVA was used for statistical analysis, followed by Tukey's post hoc test. #, no significant difference. The data are represented as means ± SEM.

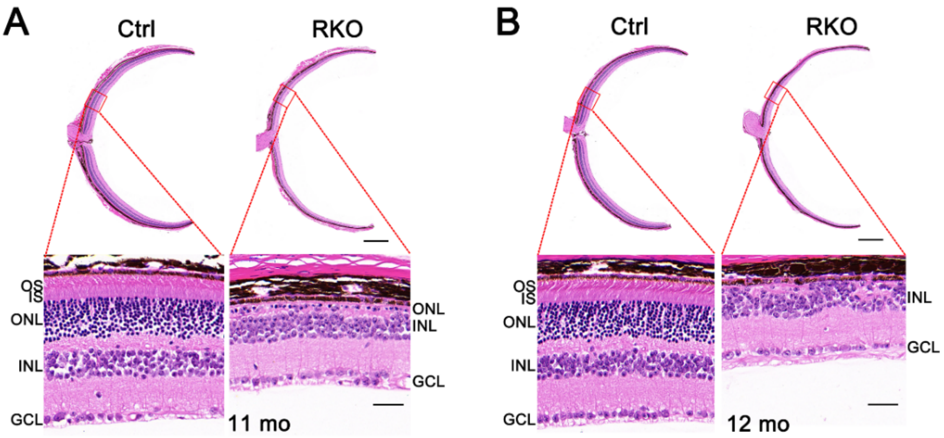

Fig. S4. Retinal ONL was almost disappeared in aged RKO mice.

(A-B) H&E staining of paraffin sections of the RKO and corresponding Ctrl retinas at the ages of 11 (A) and 12 (B) months. Scar bar: 250 μm. Higher magnification images are shown in the lower panel of each image. Scale bar: 25 μm. OS, outer segment; IS, inner segment; ONL, outer nuclear layer; INL, inner nuclear layer; GCL, ganglion cell layer.

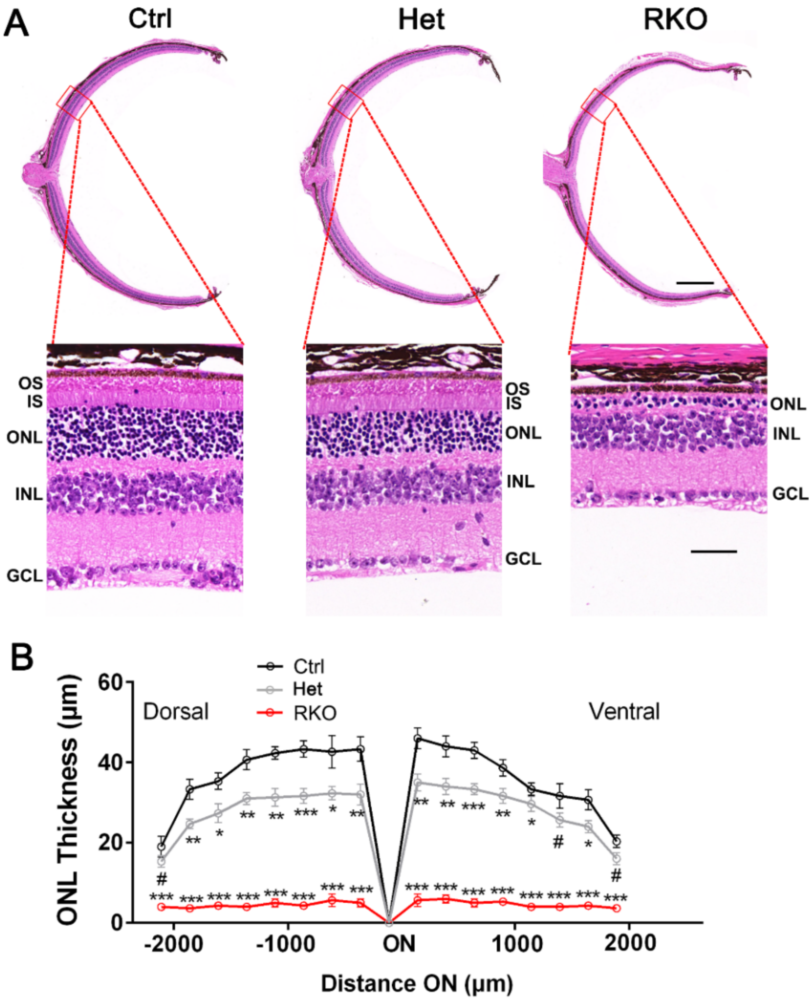

Fig. S5. Heterozygous RKO mice showed retinal degeneration at 9-month-old. (A) H&E-stained sections of retinas from 9-month-old Ctrl, heterozygous (Het) and RKO mice. Scale bar: 250 μm. Higher magnification images are shown in the lower panel of each image. Scale bar: 25 μm. (B) Quantitative assessment of the thickness of the ONL in Ctrl, Het and RKO retinas (n=7 for Ctrl and RKO animals, n=6 for Het animals). OS, outer segment; IS, inner segment; ONL, outer nuclear layer; INL, inner nuclear layer; GCL, ganglion cell layer. Two-way ANOVA was used for statistical analysis, followed by Tukey's post hoc test. *$p < 0.05$; **$p < 0.01$; ***$p < 0.001$. #, no significant difference. The data are represented as means ± SEM.

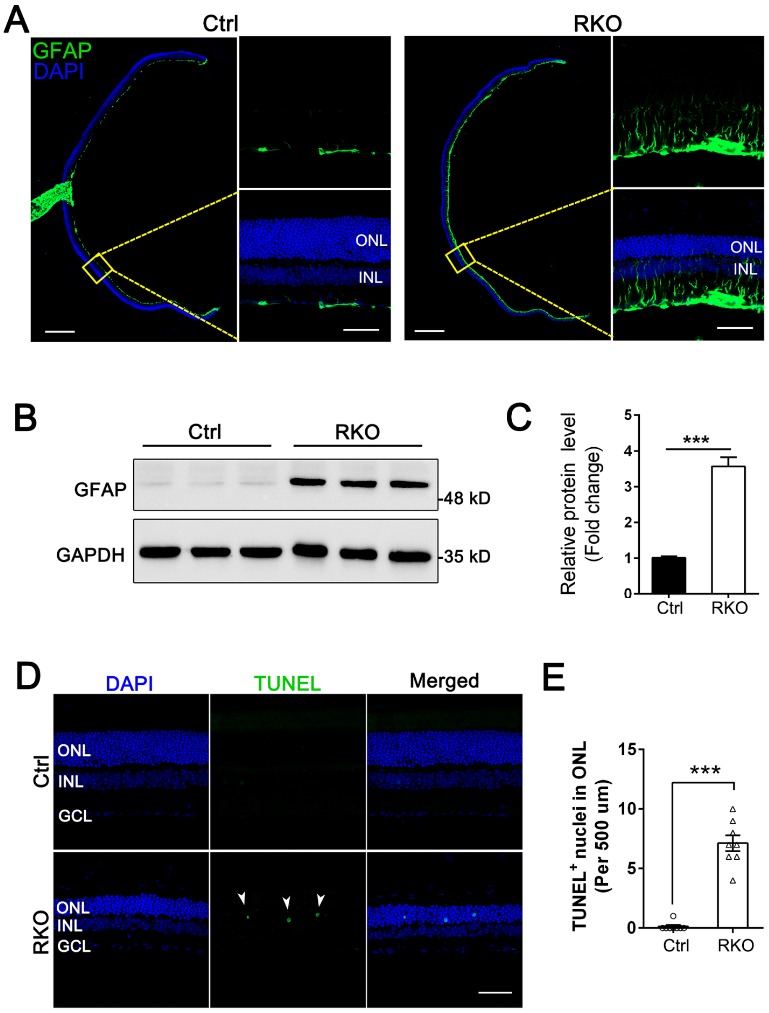

Fig. S6. Inflammatory response and apoptosis in RKO retinas.

(A) Cryosections from the retinas of 5-month-old Ctrl and RKO mice were coimmunostained for the activated astrocyte marker GFAP (green). Nuclei were counter-stained with DAPI (blue). Scale bars: 250 µm. Higher magnification images are shown in the right panel of each image. Scale bars: µm. (B-C) Representative immunoblots (B) and quantification (C) of 5-month-old retina lysates from Ctrl and RKO mice reacted with antibodies to GFAP (n=6). GAPDH was used as the loading control. (D) Immunofluorescence labelling of retina cryosections from 5-month-old Ctrl and RKO littermates with the TUNEL assay kit. Nuclei were counter-stained with DAPI (blue). TUNEL-positive cells (green) were observed in ONL of the RKO retina sections. Arrowheads represent TUNEL-positive cells. Scale bar: 25 µm. (E) Quantitative assessment of the number of TUNEL-positive cells in GCL of whole

section (n=6 for both Ctrl and RKO retinas). ONL, outer nuclear layer; INL, inner nuclear layer; GCL, ganglion cell layer. ***$p < 0.001$. The data are represented as means ± SEM.

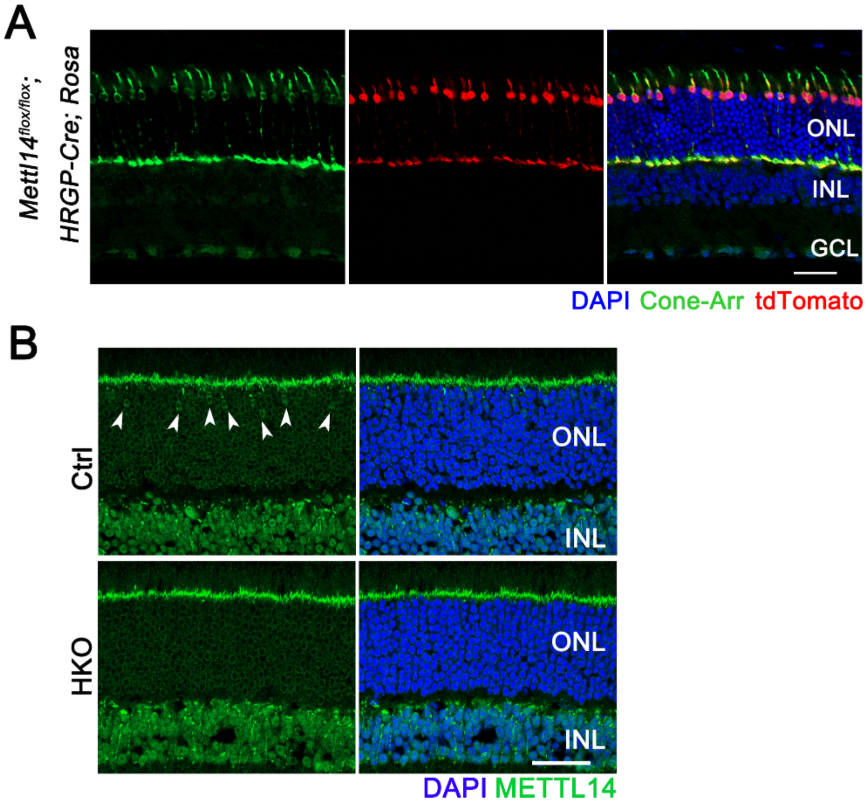

Fig. S7. Cre-mediated excision of METTL14 in HKO mice.
(A) Retina cryosections from 3-month-old *HRGP-cre; ROSA* mice were immunostained with Cone-Arrestin (Cone-Arr, green). Nuclei in cryosections were counter stained with DAPI (blue). HRGP-Cre were specificly expressed in the Cone-Arr marked cone cells (red). Scale bars: 25 µm. (B) Retina cryosections from 3-month-old HKO mice were immunostained with METTL14 (green). Nuclei in cryosections were counter stained with DAPI (blue). Arrowheads indicated that METTL14 is distinctly expressed in cone cells in control retinas, while METTL14 is absent in cones of HKO retinas. Scale bars: 25 µm. ONL, outer nuclear layer; INL, inner nuclear layer; GCL, ganglion cell layer.

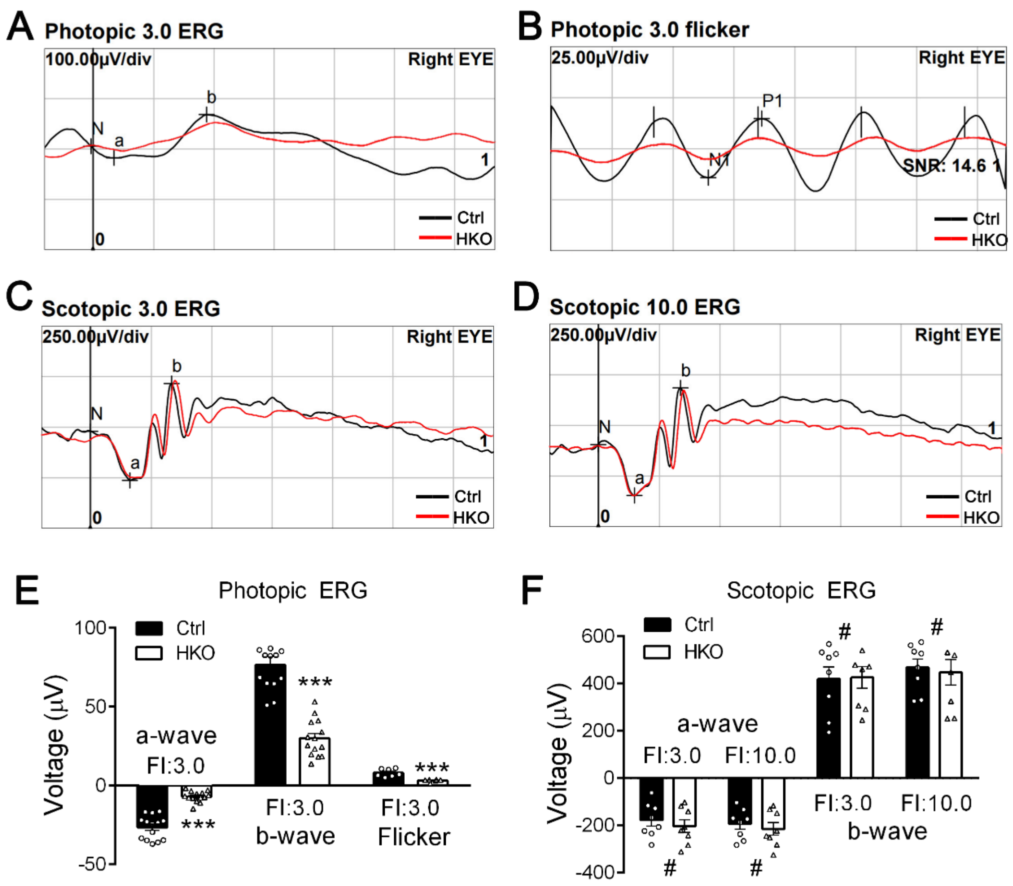

Fig. S8. Visual function analysis of HKO mice.

(A) Representative ERG traces corresponding to responses elicited by photopic conditions at flash intensities of 3 cd·sec/m^2 in control and HKO mice at 6 months of age. (B) Representative ERG traces corresponding to responses elicited by scotopic conditions at flash intensities of 3 and 10 cd·sec/m^2 in control and HKO mice at 6 months of age. (E-F) Statistical analysis was performed for the amplitudes of the a-wave, b-wave and flicker under photopic (n=14) and scotopic (n=8) conditions. Two-way ANOVA was used for statistical analysis, followed by Tukey's post hoc test. ***p < 0.001. #, no significant difference. Data are presented as the mean ± SEM.
