# Supplementary material for: Mettl14-mediated m6A modification is essential for visual function and retinal photoreceptor survival
Source: BMC Biol. 2022 Jun 13;20:140. doi: 10.1186/s12915-022-01335-x (PMC9195452; doi:10.1186/s12915-022-01335-x)
Supplement: Supplementary file 4 — Additional file 4: Tables S1. Information of selected differentially methylated RNA sites. Tables S2. Primers used in this study. Tables S3. Immunological antibodies used in this study. [file 12915_2022_1335_MOESM4_ESM.pdf]

Table S1. Information of selected differentially methylated RNA sites

Genes	Position	Start-End	Fold change	P value	Primer range	Peak range
Rho-P1	5'UTR +Exon1	chr6:115931921 -115932157	-1.504860428	2.8312 8E-07	28-141	c.1-152
Rho-P2	Exon4	chr6:115935521 -115935635	-1.753905302	8.1097 9E-07	832-932	c.822-936
Rho-P3	Exon5	chr6:115936608 -115936700	-1.837837179	2.7805 2E-07	942-995	c.936-1028
Gnat1	Exon9+3 'UTR	chr9:107675161 -107676078	-1.952391834	4.9970 4E-07	882-1043	c.862-1053
Guca1b	5'UTR+ Exon1	chr17:47385392 -47385620	-1.745925335	1.5982 5E-08	35-196	c.1-228
Pde6b-P1	5'UTR+ Exon1	chr5:108388372 -108388907	-1.947051321	1.0098 3E-07	235-431	c.1-535
Pde6b-P2	Exon2	chr5:108403319 -108403460	-1.767937887	2.2046 9E-09	538-639	c.536-676
Pde6b-P3	Exon8	chr5:108421487 -108421535	-2.21120476	1.1991 1E-06	/	c.1127-1174
Pde6b-P4	Exon12	chr5:108425232 -108425379	-1.685798505	3.2161 7E-08	1471-1547	c.1467-1581
Prph2-P1	5'UTR+ Exon1	chr17:46910458 -46911277	-1.84213758	5.0343 5E-09	92-241	c.1-580
Prph2-P2	Exon2	chr17:46919763 -46920000	-1.845250142	4.0288 4E-08	823-976	c.819-1056
Unc119	5'UTR+ Exon1	chr11:78343481 -78343720	-1.597450555	9.2427 4E-10	1-116	c.1-153
Cep164-P1	Exon3	chr9:45809703- 45809908	-1.640594889	4.6290 8E-08	442-591	c.394-598
Cep164-P2	Exon5	chr9:45802642- 45802777	-2.042014586	5.7099 8E-08	761-890	c.755-889
Cep164-P3	Exon7	chr9:45794051- 45794429	-1.955377334	2.3720 4E-07	1122-1289	c.967-1346
Cep164-P4	Exon9	chr9:45791082- 45791166	-1.935024285	1.5439 8E-08	1436-1494	c.1426-1510
Cep164-P5	Exon10	chr9:45787425- 45787511	-2.009760616	1.2620 8E-06	1538-1591	c.1511-1596
Cep164-P6	Exon16	chr9:45778407- 45778530	-1.792428947	8.8191 8E-06	2127-2247	c.2126-2250
Cep164-P7	Exon20	chr9:45775192- 45775367	-1.876793235	2.7384 2E-07	2582-2710	c.2548-2723
Fam161a	Exon3	chr11:23019999	-1.823094461	2.6985	709-872	c.491-1625

		-23021133		3E-08		
Arl3-P1	Exon4	chr19:46543481 -46543532	-1.551038535	5.9771 7E-10	/	c.263-300
Arl3-P2	Exon5	chr19:46542351 - 46542537	-1.551038535	5.9771 7E-10	330-486	c.314-501
Mast2-P1	5'UTR+ Exon1	chr4:116463736 - 116463915	-1.897659945	1.7770 6E-10	74-143	c.1-179
Mast2-P2	Exon10	chr4:116326071 -116326148	-1.86192626	2.9852 1E-10	1-37	c.926-1002
Cep164-P7	Exon20	chr9:45775192- 45775367	-1.876793235	2.7384 2E-07	2582-2710	c.2548-2723

Table S2. Primers used in this study

Number	Primer	Sequence (5' to 3')
1	Mettl14-loxP-F	AGCGGCCACTTACAGTTGAC
	Mettl14-loxP-R	CCTGTCGCCAATGGTGAATG
2	Cre-F	GAACGCACTGATTTCGACCA
	Cre-R	GCTAACCAGCGTTTTCGTTC
3	oIMR9020	AAGGGAGCTGCAGTGGAGTA
	oIMR9021	CCGAAAATCTGTGGGAAGTC
	oIMR9103	GGCATTAAAGCAGCGTATCC
	oIMR9105	CTGTTCCTGTACGGCATGG
4	Rho-cDNA-F	CTTCCCCATCAACTTCCTCA
	Rho-cDNA-R	GAATCCTCCGAAGACCATGA
5	Gnat1-cDNA-F	GAGCTTAACATGCGACGTGA
	Gnat1-cDNA-R	CGCAGTCTTTGAGGTTCTCC
6	Guca1b-cDNA-F	TTCAAGCGCTTCTTCAAGGT
	Guca1b -cDNA-R	CCTCCACAATGTCCAGGAGT
7	Pde6b-cDNA-F	AGGTCTTGGTGCGCTTTCTA
	Pde6b -cDNA-R	CGAAGGCCTCTAGGTCAGTG
8	Reep6-cDNA-F	GAGCTTAACATGCGACGTGA
	Reep6-cDNA-R	CGCAGTCTTTGAGGTTCTCC
9	Rgs9bp-cDNA-F	CGAGATGATTGACGACATGG
	Rgs9bp -cDNA-F	GCTCAGCTTTGCCACACATA
10	Rs1-cDNA-F	TCCTATGCCAGCTCTCCACT
	Rs1-cDNA-R	CACCCTCATCCTCTGTCGAT
11	Prph2-cDNA-F	ACGGACTCAAGAATGGGATG
	Prph2-cDNA-R	TAGCGATTGCTGATCCACTG
12	Unc119-cDNA-F	AAATCAAGAAGCCCCCTGTT
	Unc119-cDNA-R	CTTTTGAGGAGCTGGTTTCG
13	Rgs9-cDNA-F	GAGGATGGCATTTCTCCAAA
	Rgs9-cDNA-R	TGGGGTCTTGCAAAGGATAG
14	Wdr66-cDNA-F	ACCTTTCGGCAACACTATGG

	Wdr66-cDNA-R	AGGCCAAACAGAGTCGAGAA
15	Cep164-cDNA-F	AACCATGCCAGAACATCACA
	Cep164-cDNA-R	AACCCAGGACCAGAGGACTT
16	Fam161a-cDNA-F	TTGGCAGACATCAGAGCAGA
	Fam161a-cDNA-R	ACTCCTTCTGATGGCTTGCT
17	Cep250-cDNA-F	CCTTCCAGAAGGACAAGCTG
	Cep250-cDNA-R	TGGCGTTCTCTTTCTCCAGT
18	Crocc-cDNA-F	AGGACCGGAACACACTGAAC
	Crocc-cDNA-R	CGTTCCTCCAGCTCTTTCAC
19	Cc2d2a-cDNA-F	TGGCAGCAATCAACTGACTC
	Cc2d2a -cDNA-R	CTGCTGTGACAGTGGAGGAA
20	Arl3-cDNA-F	GACATCAGCCACATCACACC
	Arl3-cDNA-R	CTTTCTGTCGGCACTGTCAA
21	Mast2-cDNA-F	TGGCCTCATTACGTCACACT
	Mast2-cDNA-R	TCCATGTCCCTCTTTGGCAT
22	Rho-P1-F	TATGTGCCCTTCTCCAACGT
	Rho-P1-R	GAGCAGGAACATGTACGCTG
23	Rho-P2-F	CTGATCTGCTGGCTTCCCTA
	Rho-P2-R	GTGGTGAGCATACAGTTCCG
24	Rho-P3-F	GAACTGTATGCTCACCACGCT
	Rho-P3-R	TCGTCATCTCCCAGTGGATTC
25	Gnat1-P1-F	TGCCGGCAACTACATCAAAG
	Gnat1-P1-R	CGCAGTCTTTGAGGTTCTCC
26	Guca1b-P1-F	TAAGGTCAGCGGAGGACTTG
	Guca1b-P1-R	GGGACACTCCACGACAAACT
27	Pde6b-P1-F	TGCAGCACTTTTTGAACTGG
	Pde6b-P1-R	AATCCTCCAGAAGGCTGTCA
28	Pde6b-P2-F	GTCCCCACTTCAGCTCATTC
	Pde6b-P2-R	TGATCACAGCCACGACATCT
29	Pde6b-P4-F	GAAGAACTTCCAGGGCCGAC
	Pde6b-P4-R	GCTCCAGCTCTGTACACTCC

30	Prph2-P1-F	GCATCGTCCTCTTCAGCTTG
	Prph2-P1-R	TCTTCCCAGCCAGAGAGTTG
31	Prph2-P2-F	CATCAAGAGCAACGTGGATG
	Prph2-P2-R	CCAGAGGTTGAGCTCCTCAG
32	Unc119-P1-F	ATGAAGGTGAAGAAAGGCGG
	Unc119-P1-R	GACCCGGACTCAGCTTCC
33	Cep164-P1-F	AGTCCATCTGGGACCATCC
	Cep164-P1-R	TTTTGGAGGTCTCTTTGTCCTT
34	Cep164-P2-F	ACAGGGTCTCAAGGCTGCT
	Cep164-P2-R	TCATCCTCCTCGTTGGTCTC
35	Cep164-P3-F	CCTGGAACCCACAGAAAGAA
	Cep164-P3-R	CACTCTCCCTCCTGCACTCT
36	Cep164-P4-F	GAGGAAGGGCCTTTGCAGAC
	Cep164-P4-R	GTCCCTCTGCTTCCTTCCAC
37	Cep164-P5-F	CAGAGCCTCCTGAAAGCACA
	Cep164-P5-R	TTCTCCTTCTCCTCAGCGGT
38	Cep164-P6-F	CTCAGCAGCCTCCTTCGA
	Cep164-P6-R	GGCTTCGTATCGCTCTCTGG
39	Cep164-P7-F	GCCAAGAAAGAGCACACACA
	Cep164-P7-R	AATCTCTGACTCTGCGCCTG
40	Fam161a-P1-F	GACTCAGACTCCCGAACAGC
	Fam161a-P1-R	TCCCTCTTGAGCAGCTTTGT
41	Arl3-P2-F	ACTGGAGGAAGAAAAGCTGAGT
	Arl3-P2-R	GTGAGCGCTGAACAGGACT
42	Mast2-P1-F	CCGAGTTGCCCCAGCC
	Mast2-P1-R	TCCTCCAGTAGCTGCCTCC
43	Mast2-P2-F	GTCCTGGACGGTCCCCA
	Mast2-P2-R	ATTTCACTGTCAAAGGAAACTGG

Table S3. Immunological antibodies used in this study

Antibody	Dilution rate (IHC)	Dilution rate (WB)	Species	Company	Cat. No.
Anti-METTL14	1:200	1:1000	Mouse	_	_
Anti-METTL14	1:100	_	Rabbit	Abcam	ab7842
Anti-METTL3	_	1:2000	Rabbit	CST	5174
Anti-METTL3	_	1:1000	Mouse	CST	8138S
Anti-WTAP	1:200	_	Rabbit	Protein tech	15954-1-AP
Anti-GAPDH	1:200	_	Rabbit	Abcam	ab14181
Anti-GFAP	1:200	_	Mouse	Abcam	ab169276
Anti-Rhodopsin	1:200	_	Rabbit	Protein tech	11308-1-AP
Anti-Rhodopsin	_	1:2000	Mouse	CST	3700
Anti-GNAT1	1:200	1:1000	Rabbit	Abcam	ab21679
Anti-CNGA1	1:200	1:2000	Rabbit	Abcam	ab219810
Anti-PDE6B	25 μg/mL	_	_	Thermo	T13343
Anti-PRPH2	_	1:2000	Rabbit	Abcam	ab84036
Anti-GRK1	1:200	1:1000	Rabbit	Abcam	ab54460
Anti-CEP164	1:200	1:1000	Rabbit	CST	5679
Anti-Acetylated α-tubulin	1:200	1:1000	Rabbit	Abcam	ab22595
Anti-NaK ATPsae	1:500	_	Mouse	Abcam	ab18230
Anti-L/M Opsin	1:500	_	Rabbit	Sigma	AB5405
Anti-Cone Arrestin		_	Rabbit	Sigma	AB15282
Alexa Fluor™ 594 conjiaed PNA	1:200	_	_	Thermo	L32459
Anti-m6A	_	1:1000	Rabbit	SYSY	2020003
